# Large-scale analysis of post-translational modifications in E. coli under glucose-limiting conditions

**DOI:** 10.1186/s12864-017-3676-8

**Published:** 2017-04-17

**Authors:** Colin W. Brown, Viswanadham Sridhara, Daniel R. Boutz, Maria D. Person, Edward M. Marcotte, Jeffrey E. Barrick, Claus O. Wilke

**Affiliations:** 1 Institute for Cellular and Molecular Biology, The University of Texas at Austin, Austin, Texas USA; 2 Center for Computational Biology and Bioinformatics, The University of Texas at Austin, Austin, Texas USA; 3 Center for Systems and Synthetic Biology, The University of Texas at Austin, Austin, Texas USA; 4 College of Pharmacy, The University of Texas at Austin, Austin, Texas USA; 5 Department of Molecular Biosciences, The University of Texas at Austin, Austin, Texas USA; 6 Department of Integrative Biology, The University of Texas at Austin, Austin, Texas USA

**Keywords:** Post-translational modification, Proteomics, Prokaryote

## Abstract

**Background:**

Post-translational modification (PTM) of proteins is central to many cellular processes across all domains of life, but despite decades of study and a wealth of genomic and proteomic data the biological function of many PTMs remains unknown. This is especially true for prokaryotic PTM systems, many of which have only recently been recognized and studied in depth. It is increasingly apparent that a deep sampling of abundance across a wide range of environmental stresses, growth conditions, and PTM types, rather than simply cataloging targets for a handful of modifications, is critical to understanding the complex pathways that govern PTM deposition and downstream effects.

**Results:**

We utilized a deeply-sampled dataset of MS/MS proteomic analysis covering 9 timepoints spanning the *Escherichia coli* growth cycle and an unbiased PTM search strategy to construct a temporal map of abundance for all PTMs within a 400 Da window of mass shifts. Using this map, we are able to identify novel targets and temporal patterns for N-terminal N *α* acetylation, C-terminal glutamylation, and asparagine deamidation. Furthermore, we identify a possible relationship between N-terminal N *α* acetylation and regulation of protein degradation in stationary phase, pointing to a previously unrecognized biological function for this poorly-understood PTM.

**Conclusions:**

Unbiased detection of PTM in MS/MS proteomics data facilitates the discovery of novel modification types and previously unobserved dynamic changes in modification across growth timepoints.

**Electronic supplementary material:**

The online version of this article (doi:10.1186/s12864-017-3676-8) contains supplementary material, which is available to authorized users.

## Background

Post-translational modification of proteins (PTM) is a ubiquitous paradigm for dynamic cellular response and information transfer across all kingdoms of life [1]. Although historically PTM has been studied in the context of discrete and tightly-regulated signal transduction systems such as eukaryotic histone proteins [2], kinase cascades [3, 4], and prokaryotic two-component systems [5], it is only relatively recently, with the development of tandem-mass-spectrometry-based proteomics, that the abundance and complexity of PTM has become apparent [6]. A surprising result from many of these investigations has been that the phylogenetic distribution of many PTMs is much wider than had been assumed. A number of PTM types previously thought to be restricted to eukaryotic and metazoan species, such as lysine acetylation [7], serine/threonine phosphorylation [8], tyrosine phosphorylation [9, 10], and ubiquitination-like protein ligation [11], are now known to be relatively common in prokaryotic proteomes as well. This realization, in combination with the recognition that PTM plays a critical role in growth and virulence of important prokaryotic pathogens (e.g. [9, 12–16]), has highlighted the need for a better understanding of prokaryotic PTM and particularly the need for deeper, proteome-scale analysis of prokaryotic PTMs.

In response to these needs, much progress in recent years has been made in the mapping of important PTMs across a wide range of prokaryotes [1, 7, 17]. However, the vast majority of these studies are limited by only examining a handful of easily-achieved culture conditions and timepoints, and by only examining a single PTM type in isolation. The former limitation is especially important, as batch cultures grown for short time periods in rich media, as is most common for bacterial proteomics experiments, may be a poor reflection of the high-stress, nutrient-starved conditions in which bacteria spend most of their time in the wild [18, 19]. While collecting bacterial samples directly from their natural habitat is generally infeasible for proteomics experiments given the requirements for large cell numbers and pure samples, the starvation conditions commonly encountered in a bacterium’s native habitat are thought to be largely recapitulated in long-term batch culture [18, 19]. As an exponentially-growing batch culture exhausts the readily available nutrients in the growth medium, the cells undergo a regulated transition into stasis by activating a stereotypic stress response. This response usually involves a decrease of or complete stop to cell division, steep dropoffs in oxidative metabolism [20] and protein synthesis [21], sequestration of ribosomes [22, 23], activation of oxidative damage response systems [24, 25], and increased protease-mediated protein turnover [26]. Eventually, even this inactive state becomes unsustainable for the majority of cells in the culture, and a large-scale die-off takes place until the culture reaches an equilibrium where the remaining cells are able to survive on the nutrients liberated from their less fortunate culture-mates. This “deep stationary” phase of batch culture is poorly understood, but is characterized by a gradual loss of culturability (the **V**iable **B**ut **N**on **C**ulturable state [27]), likely related to accumulated cell damage, and a dynamic equilibrium of genetic changes as mutations advantageous for stationary phase growth (**G**rowth **A**dvantage in **S**tationary **P**hase, or GASP mutations [19]) are fixed by selection in the population. The low rate of protein synthesis and the potential importance of nonenzymatic protein-damage modifications in stationary phase makes an understanding of PTM chemistry and dynamics during this portion of the growth cycle especially important.

With a few very recent exceptions [28, 29], studies of PTMs at different growth phases in *E. coli* have been restricted to either a single modification or a handful of pre-specified modifications (e.g. [10, 30–32]). This limitation is largely due to both the relatively low abundance of the PTMs examined, necessitating the enrichment of modified peptides using PTM-specific antibodies [6] or chromatographic separations [33], and data analysis tools that are only useful for examining a small number of pre-specified PTMs. While enrichment is necessary for relatively transient modifications such as phosphorylation, particularly where a broad survey of targets rather than PTM dynamics is the experimental goal, it has a critical shortcoming in that it makes quantitative comparisons among PTM types, and perhaps more importantly between modified and unmodified copies of an individual protein, impossible. Adding to this problem is the fact that many of the most commonly-used software packages for MS/MS spectrum–peptide sequence matching (e.g. Mascot [34], Sequest [35], OMSSA [36], or TANDEM [37]) are limited by the need to create an in silico database of theoretical spectra using an existing peptide library; while this approach facilitates rapid searching, it makes searches involving more than a few PTM types computationally unwieldy. The spectrum of PTMs beyond a handful of well-studied examples is therefore largely unexplored.

In this work we utilize a recently developed computational tool for unrestricted analysis of PTMs in MS/MS proteomics data, MODa [38], to examine a unique proteomic dataset [39] covering 9 timepoints of the *E. coli* REL606 growth curve in minimal glucose media from early exponential growth (3 hours post-innoculation) to deep stationary phase (336h, or 2 weeks post-innoculation). MODa uses a combination of *de novo* sequence-tag matching and spectral alignment to make assigning PTM-containing spectra across a wide range of mass shifts computationally tractable, and this allows us to construct an unbiased PTM spectrum across all phases of growth for all modifications from −200 Da to +200 Da. The fine temporal resolution of our dataset then allows us to identify novel temporal trends in a number of PTMs, including N-terminal N *α* acetylation, C-terminal glutamylation, and asparagine deamidation. In addition, the lack of bias or enrichment for specific PTMs allows us to track behavior of modified and unmodified proteins across the growth cycle, and to identify a potential functional relationship between N-terminal acetylation, protein oxidative damage, and stationary-phase protein degradation.

## Results

We took advantage of a previously existing LC-MS/MS proteomics dataset [39] isolated from 3 biological replicate cultures of *E. coli* B REL606 sampled across 9 timepoints, from early exponential phase (3h post-innoculation) to extended late stationary phase (336h, or 2 weeks post-innoculation). The raw spectra from this dataset were used for simultaneous spectrum-sequence matching and PTM identification using the hybrid fragment matching/spectral alignment software MODa [38]. To reduce computation time and limit the occurrence of false positives, we restricted the MODa search to single-peptide mass shifts of +/− 200 Daltons, with one PTM allowed per peptide spectral match (PSM). To further limit the occurrence of false positive matches, we used the MODa “correct match” probability [38] to calculate the false discovery rate (FDR) and construct subsets of the highest-probability PSMs with 5 and 1% FDR (hereafter referred to as FDR5 and FDR1, respectively). The samples in our analysis were treated with iodoacetamide (IAA) to modify cysteines with a 57 Da carbamidomethyl group; during MODa analysis, this was treated as a static modification to cysteine (i.e. all modifications were relative to the molecular weight of Cys + 57Da). However, this results in an incorrect mass shift for any Cys PTMs that prevent carbamidomethylation (e.g. oxidation), so we added 57 Da to all Cys modifications to ensure that mass shifts for these modifications matched those for non-Cys residues. Note that this also results in a small number of mass shifts falling outside the +/-200 Da window specified in the initial MODa analysis, e.g. the +209 Da mass shift due to combined dithiothreitol (DTT) and carbamidomethyl modification of cysteine [40].

Localization of modifications within peptides was performed by MODa during the spectral alignment phase [38]. To most effectively combine modifications among overlapping peptides, we transformed these MODa peptide position calls into protein coordinates and used them to generate vectors of counts for all observed mass shifts at every amino acid position across the proteome. A matching set of unmodified counts was generated for all amino acid positions by counting all observations of an unmodified residue across all peptides overlapping a given amino acid position.

We identified a total of 2,527,135 PSMs across all 27 samples, corresponding to a total of 32,755 peptides that occur in at least one sample; these peptides represent 3544 individual proteins when all timepoints are considered (Table 1). FDR filtering lowers these numbers substantially, yielding 1,980,884 PSMs and 22,776 unique peptides across 2,445 proteins in the FDR5 set, and 1,473,636 PSMs and 19,265 unique peptides across 2121 proteins in the FDR1 set (Table 1). These filtered numbers are in agreement with previous proteomic experiments in *E. coli* [31, 39, 41], with the slightly lower number of proteins in our analysis, likely a result of the reduced sensitivity inherent in the larger search space used by MODa.

**Table 1:** Counts of PSMs, unique peptides, and proteins for unfiltered, 1% FDR, and 5% FDR datasets

		Total PSMs	Error Sum	FDR	Unique	Proteins
					Peptides	
Unfiltered		2,527,135	44,4225.241	0.176	32,755	3544
	Modified	608,357	19,0915.030	0.314	25,362	3478
	Unmodified	1,918,778	253,310.212	0.132	7393	66
1% FDR		1,473,636	14,736.377	0.010	19,265	2121
	Modified	198,277	3,224.088	0.016	8369	1690
	Unmodified	1,275,359	11,512.288	0.009	10,896	431
5% FDR		1,980,884	99,044.212	0.050	22,776	2445
	Modified	362,291	31,912.966	0.088	13,299	2188
	Unmodified	1,618,593	67,131.246	0.041	9477	257

We chose to focus on the FDR1 dataset for all subsequent analysis for two primary reasons. First, this was the more conservative cutoff, and by our analysis did not result in the exclusion of an excessive number of PSMs. In addition, because PSM error rates can differ significantly between modified and unmodified peptides [42, 43] we wanted to select the dataset that minimized the differences in error rates between modified and unmodified PSMs. We did observe that the distribution of MODa probabilities was in general higher for modified compared to unmodified PSMs, (Table 1, column "FDR"), but this difference was minimal in the FDR1 dataset (effective error rates of 1.6% and 0.9% for modified and unmodified, respectively).

### A large fraction of the *E. coli* proteome undergoes PTM during growth and starvation in glucose

**Fig. 1:**
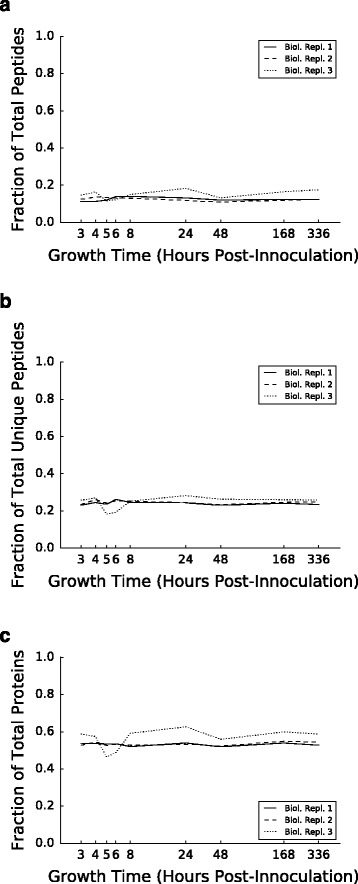
Global abundance of all modifications across growth timepoints. Shown are the fraction of total counts of PSMs (**a**), unique peptides (**b**), and proteins (**c**) containing at least one mass shift passing the 1% FDR threshold at the timepoint indicated on the *x*-axis for biological replicates 1, 2 and 3 (*solid, dashed, and dotted lines,* respectively)

Of the 1,473,636 PSMs identified across all timepoints in the FDR1 dataset, a remarkably large fraction, 198,277 (13.5%), are predicted by MODa as having a putative PTM. These modified PSMs corresponded to 8,369 out of 19,265 unique peptides (42%) having at least one modification in any sample, and 1,690 out of 2121 proteins (79.7%) having at least one modification on any constituent peptide. Interestingly, the proportion of the proteome predicted to have at least one PTM remains relatively constant across time points and biological replicates. PSMs, unique peptides, and proteins all show very little change in the proportion of overall PTM across all 9 time points (Fig. 1).

### Composition of the *E. coli* PTM spectrum

**Fig. 2:**
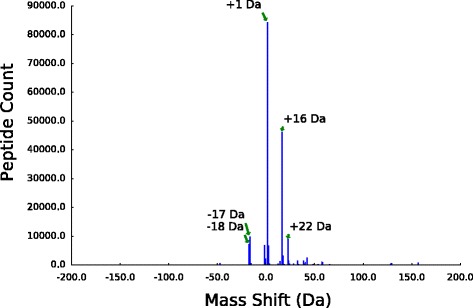
Mass-shift counts across all timepoints and datasets. Shown are the PSM counts for all mass shifts identified by MODa [38] between −200 Da and +200 Da, summed across all nine timepoints and all three biological replicates; labeled peaks are the top 5 most abundant (by raw count) mass shifts in the dataset

A unique feature of our analysis strategy is the ability to conduct an unbiased search for spectra matching post-translationally modified peptides across a wide range of possible mass shifts. We used MODa to search our raw spectral data for 400 potential peptide mass shifts, ranging from −200 Da to +200 Da; counts of PSMs for this range of mass shifts are shown in Fig. 2 and Additional file 1.

The overall abundance of individual mass shifts varies widely, with the most abundant mass shifts corresponding to small functional group modifications. The most abundant mass shift is a neutral gain of 1 Da (84,357 PSMs, 45% of all modified PSMs). In addition to simple protonation, this mass change can result from a number of more complicated modifications and MS artifacts; see Discussion. Other abundant mass shifts include oxidations (+16 Da, 46,244 PSMs, 24% of all modified PSMs; +32 Da, 1,563, 0.8% of all modified PSMs), metal ion adducts such as sodium (+22 Da, 8,882 PSMs, 4.7% of all modified PSMs) and potassium (+38 Da, 1,490 PSMs 0.79% of all modified PSMs), and neutral losses such as deamidation (−17 Da, 9,780 PSMs, 5.2% of all modified PSMs) and dehydration (−18 Da, 7,169 PSMs, 3.8% of all modified PSMs).

Commonly studied regulatory PTMs are relatively rare in our data, most likely due to their low abundance in the proteome and the fact that our samples did not undergo enrichment for specific modifications prior to analysis. Although a large number of apparent acetylations (+42 Da) were identified, only a handful of these map to known acetylated lysine residues [30, 32, 44]. A small number of phosphorylations (+80 Da) were identified, although the majority of these are modifications to an active-site serine that acts as a phosphoryl group donor during catalysis in the metabolic enzyme phosphoglucomutase (see Table 2). We also recovered a number of previously identified lysine methylation modifications for the ribosomal proteins L7/L12 (encoded by the *rplL* gene), L11 (encoded by *rplK*), and Elongation Factor Tu (encoded by *tufB*), although the last two were only observed in the first and third biological replicates, respectively. A table of counts for all mass shifts recovered by MODa is included in Additional file 2.

**Table 2:** Previously identified post-translational modifications recovered in our analysis

Locus	Position	AA	Mass Shift	PTM	Biol. Repl. 1	Biol. Repl. 2	Biol. Repl. 3	Ref.
rplL	82	K	+14 Da	Monomethylation	38 / 56 (71%)	13 / 18 (72%)	71 / 87 (81%)	[96]
tufB	57	K	+14 Da	Monomethylation	0 / 3 (0%)	0 / 6 (0%)	97 / 192 (50%)	[54, 97]
			+28 Da	Dimethylation	0 / 3 (0%)	0 / 6 (0%)	41 / 95 (43%)	
pgm	146	S	+81 Da	Phosphorylation	1/25 (4%)	0 / 11 (0%)	0 / 34 (0%)	UniProt version 2015–08 released on 2015-07-22 (UniProt consortium)
gapA	124	K	+42 Da	Acetylation	0 / 39 (0%)	1 / 71 (1.4%)	0 / 239 (0%)	[98]
	213	K	+42 Da	Acetylation	0 / 0 (0%)	0 / 3 (0%)	7 / 25 (28%)	[98]
icdA	242	K	+42 Da	Acetylation	1/198 (0.5%)	2 / 164 (1.2%)	0 / 184 (0%)	[98]
glyA	346	K	+42 Da	Acetylation	0 / 0 (0%)	1 / 1 (100%)	0 / 17 (0%)	[98]
fbaA	326	K	+42 Da	Acetylation	0 / 9 (0%)	1 / 17 (5.9%)	0 / 40 (0%)	[98]
rplK	40	K	+42 Da	Trimethylation	1 / 16 (6.3%)	0 / 1 (0%)	0 / 16 (0%)	[99]
rpsF	131	E	+129	Glutamylation	122 / 219 (55.7%)	152 / 415 (37%)	64 / 169 (38%)	[47]
secB	2	S	+42	Acetylation	184 / 221 (83%)	169 / 215 (78%)	115 / 135 (85%)	[55]
rpsE	2	A	+42	Acetylation	0 / 0 (0%)	0 / 0 (0%)	5 / 5 (100%)	[53]

### Distribution of target amino-acid residues varies widely among mass shifts

The most commonly modified amino acid across all timepoints is methionine—nearly all of these modifications are a +16 Da shift corresponding to oxidation (see Discussion)—followed by the hydrophobic amino acids Ala, Val, Leu, Ile; amide-containing amino acids Asn and Gln; and their carboxyl counterparts Asp and Glu (Table 3). The observation of a large number of modifications on amino acids with hydrocarbon side-chains, which are generally not expected to undergo PTM, can likely be explained by a combination of incorrect assignment of a mass shift to the amino acid (AA) by MODa, modification of the backbone NH or CO groups, or selection of peaks with isotopically shifted masses during MS2. The bulk of modifications to Ala, Val, Leu, and Ile are +1 Da modifications, consistent with most of these modifications being due to selection of ^13^C-containing peaks (see file “DATA_TABLE_2_mass_shifts_by_AA.tsv” provided as part of the data tables in Additional file 2). This effect is expected to occur randomly across the proteome, so the higher numbers for these particular amino acids are most likely due to their higher abundance relative to other AAs in *E. coli* proteins [45].

**Table 3:** Most commonly modified amino-acid residues

Residue	Modified PSMs	Percentage
M	42457	21.41
Q	13990	7.06
A	13682	6.90
N	13500	6.81
L	12581	6.35
V	11309	5.70
E	10872	5.48
G	10623	5.36
S	9528	4.81
T	8396	4.23
I	8267	4.17
P	8139	4.10
D	7859	3.96
K	5325	2.69
W	4964	2.50
Y	4274	2.16
C	4211	2.12
H	4084	2.06
F	3261	1.64
R	955	0.48

**Fig. 3:**
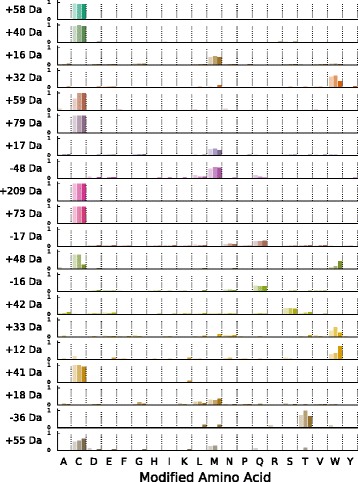
Distribution of selected mass shifts across amino acids. The height of bars within each row represents the fraction of total AA positions for the mass shift (indicated on the y axis) that were identified on each amino acid residue type (columns). Individual bars within each column represent fractions for each biological replicate (replicates 1, 2 and 3 from left to right within each column). Mass shifts are ordered by the single-AA bias score (the ratio of counts for the most commonly modified AA type to the mean of the counts for all other types; see Methods) with the highest score (most biased for a single AA) at the top; only the top 20 mass shifts are shown. Note that a constant mass shift of +57 Da was added to all cysteine modifications to correct for the presence of carbamidomethylation, meaning that a small number of cysteine modifications (e.g. +209 Da) fall outside of the mass range scanned by MODa (+/−200 Da)

We constructed the distribution of targeted amino acids for each mass shift by counting occurrences of each mass shift–AA pair across all nine time points. We observed significant differences among mass shifts in preference for a single type (or, in some cases, groups) of amino acid residues; the +22 Da and +38 Da modifications, for example, show a broad distribution across AA types, while +16 Da and −2 Da show strong (though not exclusive) preference for methionine. To quantify these differences in AA distribution, we ranked mass shifts by the ratio: PSMs for most common AA / mean(PSMs for all other AAs). AA distributions for the the top ranked (most biased towards one AA across multiple biological replicates) mass shifts are shown in Fig. 3.

A large number of modifications with a strong preference for cysteine residues were identified in all three biological replicates; most of these are likely artifacts of IAA treatment during sample preparation, and correspond to common modifications co-occuring with carbamidomethylation (+57 Da), e.g. +58 Da (57 + 1 Da), +59 Da (57 + 2 Da), and +40 Da (57 - 17 Da). The +209 Da mass shift, corresponding to a carbamidomethylated dithiothreitol modification of the cysteine thiol group, is a minor artifact of the reduction and alkylation of cysteine during sample preparation [40]. The +48 Da mass shift was almost exclusively found at catalytic cysteine residues in a handful of proteins, and corresponds to the hyperoxidation of the cysteine thiol group (Cys-SH) into cysteic acid (Cys-SO_3_H). This modification is likely to be inactivating and irreversible, resulting in the increased accumulation of the modified form throughout the stationary phase. Among modifications targeting non-Cys residues, putative oxidative modifications show the strongest bias towards a single AA, with the +32 Da and +16/+17 Da modifications showing strong preferences for tryptophan and methionine, respectively. The eighth-ranked −48 Da modification is likely also a result of oxidation via dethiomethylation of methionine residues [46]. The strong preference of the acetylation mass shift (+42 Da) for serine is largely due to modifications on protein N-termini (see Section “N-terminal and C-terminal modifications”). A table of counts for each mass shift-amino acid pair is included in Additional file 2.

### N-terminal and C-terminal modifications

**Fig. 4:**
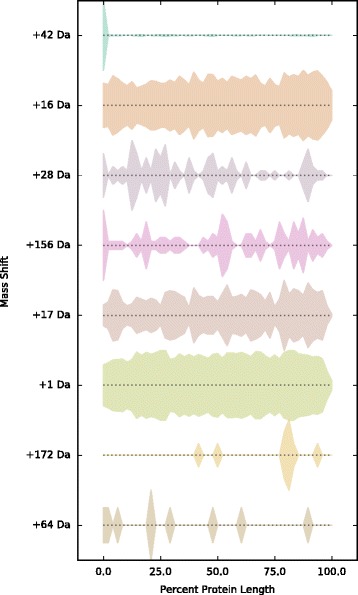
Distribution of Nt-biased mass shifts across positions in protein sequence. The widths of traces within each row represent the density of unique positions identified for the mass shift indicated to the left along target proteins, normalized by protein length (*x*-axis). Traces are plotted symmetrically about the *x*-axis. Mass shifts are ranked from *top to bottom* by combined *p*-value from the Fisher’s exact test for N-terminal modification enrichment across all three replicates (see section “N-terminal and C-terminal Modifications” and Table 4), with mass shifts having the strongest N-terminal enrichment at the top

**Fig. 5:**
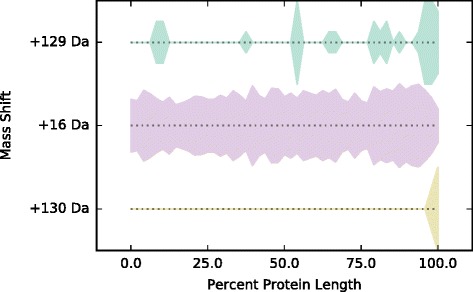
Distribution of Ct-biased mass shifts across positions in protein sequence. The widths of traces within each row represent the density of unique modified positions (i.e. positions with more than one modified PSM; each position is counted once per protein) identified for each mass shift (indicated to the *left*) along target proteins, normalized by protein length (*x*-axis). Traces are plotted symmetrically about the *x*-axis. Mass shifts are ranked from *top to bottom* by combined *p*-value from the Fisher’s exact test for N-terminal modification enrichment across all three replicates (see section “N-terminal and C-terminal Modifications” and Table 5), with mass shifts having the strongest C-terminal enrichment at the top

To search for modifications that preferentially occur at protein N and C termini, we used Fisher’s exact test (FET) to compare the ratio of modified : unmodified counts of each mass shift occurring at the N or C terminus of a protein to the same ratio for mass shifts occurring at all other positions. FET *p*-values for N-terminal and C-terminal enrichment were calculated for all mass shifts within each biological replicate and filtered for consistency by requiring all three replicates to have *p*<0.05. Nt- and Ct-biased mass shifts are shown in Tables 4 and 5. We also examined the distribution of unique modified positions for these Nt- and Ct-biased mass shifts as a function of normalized protein length, to determine whether the observed positional bias was a general feature of the mass shift or due to a small number of highly abundant modified positions (Figs. 4 and 5).

**Table 4:** Mass shifts occurring more frequently on N-terminal ends of proteins

Mass Shift	Biol. Replicate	N-terminal	Non-N-Terminal	*p*-value
+42 Da	1	759 / 1869 (40.61%)	132 / 7860 (1.68%)	0.0
	2	657 / 1561 (42.09%)	111 / 6814 (1.63%)	0.0
	3	682 / 1930 (35.34%)	147 / 7681 (1.91%)	0.0
+16 Da	1	1187 / 8016 (14.81%)	13633 / 510869 (2.67%)	0.0
	2	972 / 6971 (13.94%)	9350 / 385682 (2.42%)	0.0
	3	531 / 5216 (10.18%)	20571 / 423856 (4.85%)	7.36×10^−55^
+28 Da	1	16 / 128 (12.50%)	54 / 31261 (0.17%)	4.56×10^−24^
	2	27 / 136 (19.85%)	11 / 28221 (0.04%)	1.78×10^−55^
	3	45 / 140 (32.14%)	305 / 33420 (0.91%)	1.65×10^−54^
+156 Da	1	20 / 1051 (1.90%)	277 / 54832 (0.51%)	1.17×10^−6^
	2	14 / 832 (1.68%)	154 / 58376 (0.26%)	1.34×10^−7^
	3	26 / 674 (3.86%)	313 / 43231 (0.72%)	2.73×10^−11^
+17 Da	1	60 / 3107 (1.93%)	585 / 178417 (0.33%)	8.94×10^−26^
	2	45 / 2730 (1.65%)	512 / 116280 (0.44%)	6.38×10^−13^
	3	54 / 2070 (2.61%)	1960 / 147203 (1.33%)	7.64×10^−6^
+1 Da	1	138 / 6868 (2.01%)	27945 / 2042671 (1.37%)	1.88×10^−5^
	2	151 / 5934 (2.54%)	25111 / 1455121 (1.73%)	5.28×10^−6^
	3	204 / 6226 (3.28%)	30808 / 1810086 (1.70%)	1.52×10^−17^
+172 Da	1	15 / 15 (100.00%)	0 / 9431 (0.00%)	3.11×10^−48^
	2	1 / 1 (100.00%)	1 / 11114 (0.01%)	1.8×10^−4^
	3	14 / 14 (100.00%)	11 / 7365 (0.15%)	2.77×10^−37^
+64 Da	1	13 / 58 (22.41%)	13 / 1620 (0.80%)	1.84×10^−13^
	2	4 / 48 (8.33%)	6 / 1340 (0.45%)	2.27×10^−4^
	3	13 / 103 (12.62%)	19 / 2119 (0.90%)	3.58×10^−10^

**Table 5:** Mass shifts occurring more frequently on C-terminal ends of proteins

Mass Shift	Biol. Replicate	C-terminal	Non-C-terminal	*p*-value
+129 Da	1	165 / 703 (23.47%)	78 / 10189 (0.77%)	2.00×10^−142^
	2	212 / 886 (23.93%)	49 / 8541 (0.57%)	1.98×10^−177^
	3	82 / 502 (16.33%)	59 / 8478 (0.70%)	4.36×10^−67^
+16 Da	1	39 / 205 (19.02%)	14781 / 518680 (2.85%)	7.88×10^−21^
	2	42 / 245 (17.14%)	10280 / 392408 (2.62%)	7.76×10^−22^
	3	22 / 103 (21.36%)	21080 / 428969 (4.91%)	5.13×10^−09^
+130 Da	1	56 / 427 (13.11%)	2 / 366 (0.55%)	1.18×10^−14^
	2	75 / 542 (13.84%)	0 / 549 (0.00%)	1.02×10^−24^
	3	18 / 276 (6.52%)	0 / 196 (0.00%)	8.29×10^−05^

Eight mass shifts were identified as Nt-biased after filtering (Table 4 and Fig. 4). The strongest Nt preference is displayed by the +42 Da mass shift, corresponding to N-terminal acetylation, with modified N termini representing 35–42% of total observed counts for positions with at least one +42 Da count. The remaining Nt-biased mass shifts fall into two broad categories. The first are rare modifications that occur at a small number of positions at high frequency, such as the +28 Da mass shift (possible retention of formylation on an Nt-terminal fMet, 12–32%), the +64 Da mass shift (possible modification by acetate, 8–22%) and the +172 Da mass shift (100% in all replicates). The second category is comprised of common modifications that occur at low frequency across a larger number of positions; this includes oxidation (+16 Da, 10–14%), most commonly of a retained Nt methionine, and protonation (+1 Da, 2–3%). A beneficial feature of our analysis is the ability of MODa to identify modified N-terminal residues even in the presence of un-annotated N-terminal methionine cleavages. For the protein SecB, for example, we recovered abundant N-terminal peptides which had both undergone N-terminal Met cleavage and putative acetylation at the penultimate N-terminal Ser residue (see Additional file 3), despite the fact that this protein had not been annotated as having its N-terminal Met cleaved in the UniProt database.

Only three mass shifts were identified as Ct-biased after consistency filtering (Table 5 and Fig. 5). Two of these, +129 Da (16–24% of counts at C-terminal positions across the three replicates modified, compared to < 1% of counts at all other positions) and +130 Da (6.5–14% of counts at C-terminal positions across the three replicates modified, compared to < 1% of counts at all other positions), most likely correspond to the same modification, C-terminal addition of a glutamate residue. Interestingly, the third C-terminal mass shift is oxidation (+16 Da), which is observed to occur at high frequency (17–20% modified counts across replicates at C-terminal residues with at least one +16 Da modification, compared to 2.6–5% at all other modified positions) on C-terminal residues as well as N-terminal residues, although the C-terminal modification is observed for a smaller set of proteins.

The C-terminal glutamylation modification is especially interesting. The most frequent target for this modification is the C terminus of the 30S ribosomal protein S6 (RpsF), which is known to undergo post-translational modification with 1–4 glutamate residues (mass = 129 Da) [47]. The enzymatic addition of these Glu residues to S6 proceeds in a stepwise fashion, and any modification of two or more Glu residues would fall outside the range of mass shifts that were considered in our analysis, so it is likely that the mono-glutamylated S6 we observed only represents a subset of the total modified S6 present in our samples.

We also identified a previously unreported C-terminal +129 Da modification of the stationary phase ribosomal stability factor RaiA / YfiA [22]. YfiA binds within the mRNA tunnel of the 30S subunit [48, 49], where it inhibits translation [48, 50] and prevents subunit dissociation and 100S dimer formation for a subset of ribosomes in stationary phase [51]. YfiA and S6 lie near one another within the 30S subunit, and both proteins’ C termini extend towards the same region of the 16S rRNA on the subunit surface (Additional file 4), although the modified C-terminal tails themselves are not resolved in the crystal structure. The temporal modification patterns of S6 and YfiA differ dramatically (Additional file 5). S6 levels of both total PSM counts and Ct +129-Da modified counts peak in mid-exponential phase, followed by a steep drop to a lower number of counts that is maintained through late stationary phase; the relative proportion of +129 Da modified counts remains nearly unchanged across all time points. In contrast, YfiA shows low or no counts of either modified or unmodified PSMs until the onset of stationary phase, when overall counts increase dramatically, accompanied by a low but constant level of C-terminal +129 Da modification through late stationary phase. The exponential phase enrichment we observed for the +129 Da mass shift is therefore due largely to changes in overall expression of its target proteins rather than differential modification.

### Temporal patterns

The glucose starvation dataset used in our analysis is unique in the wide range of timepoints (3h–336h) that were sampled. Changes in abundance during different phases of the growth cycle in liquid culture have been observed for individual PTMs, but an unbiased examination of temporal variation in the global PTM profile has not been performed in *E. coli*. To identify mass shifts with significant frequency changes over the growth cycle, we first pooled four of our nine time-point samples into exponential-phase samples (3h, 4h, 5h, and 6h, EXP) and four into stationary-phase samples (24h, 48h, 168h, 336h, STA). (We did not include the 8h sample in this analysis.) We then grouped counts across modified amino-acid positions by mass shift–AA pairs and compared the ratio of modified:unmodified counts at all modified positions in the EXP and STA pools using Fisher’s exact test (FET) [52]. Mass shift–AA pairs were called as significant if their FET *p*-values passed a false-discovery rate filter (< 5% FDR by the Benjamini-Hochberg step-down procedure) in all three biological replicates. Because we used a two-tailed test that was unable to determine the direction of enrichment (i.e., EXP > STA or EXP < STA), we subsequently divided significant mass shift-AA pairs into EXP > STA or EXP < STA groups using the FET log-odds score.

**Fig. 6:**
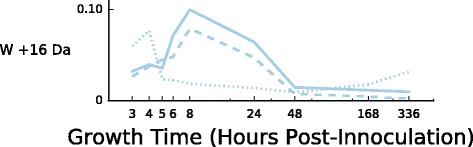
Abundance across all growth timepoints of tryptophan monooxidation, the sole mass shift with stronger modification in exponential phase. The plot shows the fractional modification *N*_mod_/(*N*_mod_+*N*_unmod_) across all nine time points, for positions having at least one W +16 Da modification at any time point. Individual traces show results for individual biological replicates 1 (*solid lines*), 2 (*dashed lines*), and 3 (*dotted lines*)

We identified only a single mass shift that consistently shows significantly higher levels of modification in exponential phase across all three biological replicates, a +16 Da modification of tryptophan (3.78–4.33% of total counts at modified positions across the three biological replicates have the mass shift in exponential phase, 1.60–1.69% in stationary phase, Table 6). The behavior of this mass shift differs slightly across the three biological replicates: in biological replicates 1 and 2, the +16 Da Trp modification shows a spike in abundance near the Exponential-Stationary phase transition (8h), followed by a drop to near zero by mid-stationary phase (48h), while replicate 3 shows a spike of enrichment earlier in exponential phase (4h) followed by a steep drop off at the 5h timepoint (Fig. 6).

**Table 6:** Mass-shift–amino-acid pairs with elevated frequency in exponential phase

Mass	Amino	Biol.	Exponential	Stationary	*p*-value
Shift	Acid	Replicate			
+16 Da	W	1	285/6836 (4.17%)	248/8775 (2.83%)	5.55×10^−06^
		2	238/6182 (3.85%)	141/7242 (1.95%)	4.31×10^−11^
		3	282/6182 (4.56%)	113/6414 (1.76%)	9.13×10^−20^

**Fig. 7:**
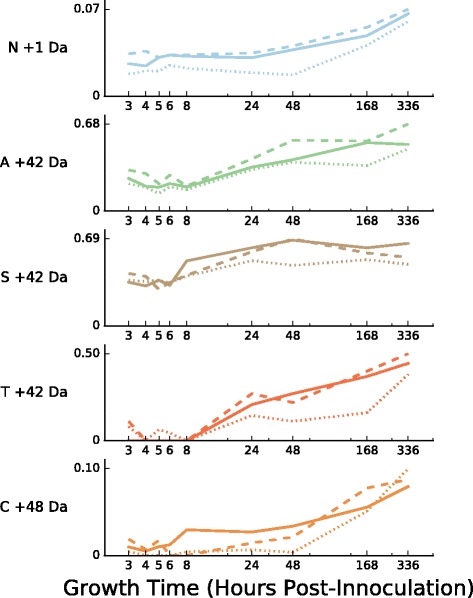
Abundance across all growth timepoints of mass shifts with stronger modification in stationary phase. Each plot shows the fractional modification *N*_mod_/(*N*_mod_+*N*_unmod_) across all nine time points, for positions having at least one modification of the indicated type at any time point. Individual traces within each plot show results for individual biological replicates 1 (*solid lines*), 2 (*dashed lines*), and 3 (*dotted lines*). Mass shift are ranked from top to bottom by *p*-value from the Fisher’s exact test for modification enrichment in exponential phase (STA>EXP; see Section “Temporal patterns” and Table 7), averaged across all three replicates, with the most stationary-phase-enriched (lowest *p*-values) at the *top*

We identified five mass shifts that consistently show significantly higher levels of modification in stationary phase across all three biological replicates: a +1 Da modification of asparagine (1.90–3.06% of total counts at modified positions have the mass shift in exponential phase, 2.95–4.60% in stationary phase); +42 Da modifications of serine, alanine, and threonine (29.78–31.71% EXP, 46.30–60.07% STA; 18.33–22.45% EXP, 34.81–46.46% STA; and 0.0–3.37% EXP, 9.46–15.70%, respectively), and a +48 Da modification of cysteine (0.94–1.09% EXP, 3.11–4.19% STA) (Table 7). As with the exponential-phase-biased mass shifts, we observed different temporal patterns when timepoints are considered individually (Fig. 7). For example, the +1 Da asparagine modification and the +48 Da cysteine modification show steady increases across stationary phase, reaching their highest value at the latest stationary phase timepoint (336h), while the +42 Da modification to serine shows a more step-like increase in abundance near the onset of stationary phase, with abundance remaining fairly constant through the latest timepoints.

**Table 7:** Mass-shift–amino-acid pairs with elevated frequency in stationary phase

Mass shift	Amino acid	Biol. replicate	Exponential	Stationary	*p*-value
+1 Da	N	1	1273/45738 (2.78%)	2260/52371 (4.32%)	2.49×10^−38^
		2	1133/31975 (3.54%)	1663/36568 (4.55%)	3.02×10^−11^
		3	988/40658 (2.43%)	1313/43285 (3.03%)	8.55×10^8^
+42 Da	S	1	176/585 (30.09%)	322/534 (60.30%)	2.08×10^−24^
		2	143/461 (31.02%)	287/509 (56.39%)	2.02×10^−15^
		3	195/597 (32.66%)	269/570 (47.19%)	4.77×10^−07^
+42 Da	A	1	55/261 (21.07%)	59/135 (43.70%)	4.15×10^−06^
		2	55/202 (27.23%)	68/125 (54.40%)	1.22×10^−06^
		3	38/210 (18.10%)	63/165 (38.18%)	2.08×10^−05^
+48 Da	C	1	15/1604 (0.94%)	51/1217 (4.19%)	1.18×10^−08^
		2	14/1285 (1.09%)	30/770 (3.90%)	4.80×10^−05^
		3	1/964 (0.10%)	22/707 (3.11%)	6.93×10^−08^
+42 Da	T	1	0/244 (0.00%)	57/363 (15.70%)	3.54×10^−14^
		2	5/263 (1.90%)	51/321 (15.89%)	1.31×10^−9^
		3	11/326 (3.37%)	40/423 (9.46%)	1.11×10^−3^

### Preferential persistence of N-terminally acetylated proteins in stationary phase

The N-terminal bias and preference for serine, alanine, and threonine residues observed for the +42 Da mass shift strongly suggests that this modification corresponds to N-terminal N *α*-acetylation. Although cotranslational N-terminal N *α* acetylation (NtAc) is widespread in eukaryotic proteins, the prevalence and physiological significance of this modification in prokaryotes is poorly understood. In *E. coli*, only five native proteins are known to possess an NtAc modification: the ribosomal proteins S5 (encoded by the *rpsE* gene), S18 (encoded by the *rpsR* gene), and L12/7 (encoded by the *rplL* gene)[53]; elongation factor Tu (EFTu, encoded by the *tufB* gene) [54]; and the chaperone SecB [55]. In addition, a number of heterologous eukaryotic proteins are modified with an NtAc when overexpressed in *E. coli* [56–59].

We identified 44 Nt-acetylated proteins (Additional files 2, 3, 6, and 7) and were able to recover modified peptides from known Nt-acetylation target SecB (Additional file 3) and a small number of peptides matching Nt-acetylated ribosomal protein S5 (Additional file 7) in our initial MODa dataset. The low peptide counts for S5, as well as the absence of modified PSMs for the other known (and highly abundant) targets ribosomal proteins S18 and L7/12, as well as EFTu, are likely due to the presence of tryptic cleavage sites within a few residues of the N-terminus in all three of these proteins (Nt-AHIE**K**QAGE for S5, Nt-A**R**YF**RRRK**F for S18, Nt-SIT**K**DQIEE for L7/12, and Nt-S**K**E**K**FERT**K** for EFTu). This means that most copies of the protein present in our samples will produce N-terminal peptides too short to recover during subsequent liquid chromatography and MS/MS steps. Consistent with this interpretation, we were able to recover abundant peptides from non-N-terminal regions of all four of these proteins, and the small number of S5 N-terminal peptides that were recovered were all the result of missed cleavage events at the N-terminal-most cleavage site. Among the NtAC peptides that were recovered in our modA dataset, the Nt fragment from SecB is by far the most frequently observed, representing 15–41% of the total Nt-Acetylated peptides across the nine time points. In addition, six other proteins from our dataset were previously identified as Nt-acetylation targets in an enrichment-based analysis of N-terminal modifications in *Pseudomonas aeruginosa* [13] (see Table 8).

**Table 8:** Overlapping N-terminal N *α*-acetylation targets between current data and *P. aeruginosa* [13]

*E. coli*	Locus	*E. coli* Peptide	*P. aeruginosa*	*P. aeruginosa* Peptide	Description
Locus Tag			Locus Tag		
ECB_00686	sucB	SSVDILVPDLPESVADATVATWHKK	PA14_44000	MAIEIK	Dihydrolipoamide Succinyltransferase
ECB_03391	dppF	STQEATLQQPLLQAIDLKK	PA14_58490	METVLTAR	Dipeptide transporter ATP-binding subunit
ECB_00915	rpsA	TESFAQLFEESLKE	PA14_23330	SESFAELFEESLK	30S ribosomal protein S1
ECB_00155	yadR	SDDVALPLEFTDAAANKV	PA14_08510	SIETFTPTPLLFTPGAANK	Iron-sulfur cluster insertion protein ErpA
ECB_00183	accA	SLNFLDFEQPIAELEAKI	PA14_23860	SNWLVDKLIPSIMR	Acetyl-CoA carboxylase carboxyltransferase subunit alpha
ECB_03467	secB	SEQNNTEMTFQIQRI	PA14_67720	TEQATNGAADEQQPQFSLQR	Preprotein translocase subunit SecB

**Fig. 8:**
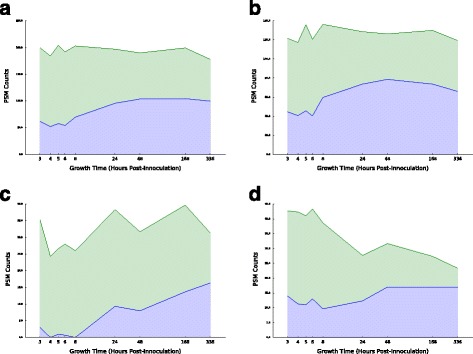
N-terminal +42 Da modified proteins are preferentially retained in stationary phase. Plots show unmodified (*green*) and +42 Da modified (*blue*) PSM counts for all N-terminal positions possessing at least one +42 Da modification at any time point, averaged across the three biological replicates. Shown are total counts (**a**), counts for peptides with a penultimate (i.e. following a cleaved N-terminal methionine) serine residue (**b**), a penultimate threonine residue (**c**), and a penultimate alanine residue (**d**)

We observed that NtAc modified proteins are proportionally more heavily modified in stationary phase (Fig. 7). This pattern could be explained by (i) an increase in acetylation activity in stationary phase and/or (ii) a proportionally larger decrease in non-acetylated copies of a protein relative to acetylated copies in stationary phase. To differentiate between these scenarios, we plotted total PSM counts and NtAc-modified PSM counts for pooled NtAc-targeted proteins across all nine time points (Fig. 8). When all NtAc-targeted proteins are considered (Fig. 8, top left panel), the total number of PSMs stays appoximately constant, while the number of NtAc-modified PSMs increases by nearly twofold in early stationary phase, consistent with scenario (i). However, NtAc-targeted proteins pooled by penultimate amino acid (Fig. 8) or individual NtAc-targeted proteins (Additional files 3, 6 and 7) show a mixture of both scenarios. NtAc-targeted proteins with a penultimate serine or threonine residue, for example, exhibit a pattern consistent with scenario (i), similar to the pattern for all targets (Fig. 8, top right and bottom left panels). Proteins with a penultimate alanine, however, show a slight increase in modified peptides at the onset of stationary phase, accompanied by a large drop in unmodified peptides (Fig. 8, top right panel). Many of the the most heavily NtAc-modified proteins also show this pattern, such as LysS, SpeA, PdxH, and SecB (Additional file 3), and IlvA and KdgR (Additional file 7). This preferential retention of NtAc-modified peptides in stationary phase suggests that NtAc may play role in protein stability by acting as an anti-degradation signal (see Discussion). A table of all Nt-acetylation sites recovered by MODa is included in Additional file 2.

### Asparagine deamidation is strongly enriched in very late stationary phase

An interesting temporal pattern was also identified for the +1 Da modification of Asparagine residues, which increases in frequency throughout stationary phase and peaks at the last timepoint (336h) (Fig. 7 and Additional file 8). A +1 Da modification occurring on an asparagine residue is known to be a signature of nonenzymatic asparagine deamidation, in which a backbone nitrogen initiates a nucleophilic attack on the amide carbon of the asparagine side chain (or the asparagine amide nitrogen on the backbone carbonyl carbon) to form a cyclic succinimide intermediate [60–62]. This intermediate can then resolve by hydrolysis to either convert the original asparagine to an aspartate residue, or rearrange to form an isopeptide linkage through isoaspartate; both of these events result in a +1 Da mass shift.

We found that +1 Da modifications were the most frequently observed modification in our dataset. They likely result from a variety of sources, most importantly isotopic mass shifts from ^13^C-containing peptides. While our dataset certainly contains peptides with +1 Da modifications resulting from isotopic peak shifts, two observations support our hypothesis that a significant fraction of +1 Da modifications to Asp are due to deamidation. First, +1 Da modifications from isotopic mass shifts are expected to be more prevalent on peptides with higher *m*/*z* values. There is indeed a general correlation between peptides with high *m*/*z* values and peptides called by MODa as having a +1 Da mass shift; while the median *m*/*z* value for all peptides is 808.04, that for +1 Da modified peptides is 1001. However, while median *m*/*z* values for peptides with +1 Da modifications to all AAs except cysteine vary between 980 and 1065, the Asn +1 Da peptides stand out as having a lower median of 931.4, and have an *m*/*z* distribution that is significantly different from the overall +1 Da *m*/*z* distribution (Kolmogorov-Smirnov test, *p*=2.48×10^−251^). This finding is consistent with Asn +1 Da peptides being a mixture of ^13^C-peak selection artifacts and genuine Asn deamidation modifications. In addition, high-PSM-count Asn +1 Da modifications, but not +1 Da modifications to other AA types, are enriched for Glycine, Serine, and Asparagine residues at the amino acid position following the modified Asn (Additional file 9), a pattern that is consistent with known sequence preferences for Asn deamidation [63].

Although asparagine deamidation can occur spontaneously as an experimental artifact during preparation of proteomic samples [64], a number of lines of reasoning suggest that at least a subset of the modifications we observe were present in the samples prior to processing. First, we observe a nearly identical pattern of increasing Asp +1 Da modification across all three of our biological replicates (Fig. 7). All timepoints were collected from a single set of cultures started on the same day, each biological replicate was grown independently (on a different day) from the others, and all timepoints from a single replicate were processed for proteomic analysis in parallel. The bulk of nonenzymatic deamidation during proteomic sample prep has been shown to occur during tryptic digest [64], with both longer incubation time and basic pH increasing the occurrence of deamidated peptides. The samples used to generate our data were treated with a short tryptic digest (5h) in the presence of near-neutral buffer (50mM Tris, pH 8.0), conditions that should limit spontaneous asparagine deamidation. In addition, both pH and incubation time should be identical across all timepoints (because samples were processed together), and any nonenzymatic deamidation should therefore also be constant across timepoints. The pattern does not appear to be explained simply by increased expression of modified proteins during stationary phase, as the pattern is observed even for individual modifications that have high abundance in both exponential and stationary phases (Additional file 8). A table of all putative asparagine deamidation sites recovered by MODa is included in Additional file 2.

### Oxidative modifications of methionine and tryptophan are variable across biological replicates

Oxidation (+16 Da) modifications, particularly of methionine, are very common in our data, but with the exception of +16 Da modification of tryptophan residues (Fig. 6), oxidations in general are not identified as having a significant bias for either exponential or stationary phase. Both Met +16 Da and Trp +16 Da show significant variability among the three biological replicates, with replicates one and two showing a similar pattern of relative modification enrichment over time, while replicate three has a different pattern (Additional files 10 and 11). In addition, for both modifications replicates one and two show a peak of modified peptide counts centered at or near the 8 h time point (the exponential-stationary phase transition; this timepoint was excluded from our initial comparisons of stationary vs. exponential enrichment), with the proportion of modified PSMs then decreasing to early-exponential-phase levels or below by 24 h.

The reason for the discrepancy between the third replicate and the two others is unclear; the third replicate was prepared at a later date than the first two replicates, so it is likely that much of this variability is due to batch effects. This observation in combination with the common occurrence of oxidative modifications as experimental artifacts [65] makes it difficult to draw any biological conclusions from the temporal patterns of oxidative modifications. Because all samples within each replicate were prepared in parallel, we would expect any artifactual modifications to covary across samples in a replicate; our observation of within-replicate variance correlated across at least two samples is therefore difficult to explain unless some samples have a higher intrinsic rate of artifactual oxidation, or some amount of genuine biological variation is present.

We observe the discrepancy among replicates only for oxidative modifications and not for other modified peptide counts or overall peptide levels, so one possibility is that a difference in redox conditions in sample processing influenced the number of oxidized peptides that were recovered. Differential modification in the third replicate is apparent in the temporal modification patterns of individual target sites (Additional files 12 and 13), but does not display a consistent pattern across sites. Tables of all methionine and tryptophan oxidation sites are included in Additional file 2.

## Discussion

We have leveraged a large proteomics dataset [39] and the fast multi-blind spectral alignment algorithm MODa [38] to construct a comprehensive, unbiased map of all protein post-translational modifications between −200 and +200 Da at 9 timepoints, spanning early exponential phase (3h post-innoculation) through late stationary / starvation phase (336h, or 2 weeks post-innoculation). From this map, we have identified post-translational mass shifts with statistically significant differences in modification stoichiometry between N- and C-terminal ends of proteins and between exponential and stationary phases. This analysis has enabled us to identify previously unobserved temporal patterns and novel target proteins for known modifications, and to identify possible novel modifications. Finally, by comparing temporal patterns of modified and unmodified PSM counts for individual AA positions, we have been able to identify a possible relationship between post-translational modification and protein degradation rate in stationary phase.

Although decades of work have been dedicated to studying the biochemical and physiological function of post-translational modifications, much of this work has focused on a handful of modification chemistries such as Ser/Thr phosphorylation and Lys acetylation. Technical limitations in instrument sensitivity, sample preparation, and data analysis have meant that even these well-studied PTMs are often studied in isolation, and their place in the overall context of the cell, in terms of the overall set of pathways and proteins that utilize them, their interaction with other modifications, and their abundance relative to other modifications, is lost. An intriguing feature of our dataset is the relative scarcity of the most commonly studied regulatory modifications, such as phosphorylation and acetylation; in the few cases where such modifications are identified, they tend to occur at low frequency, even on very abundant proteins (Table 2).

The overall shape of the *E. coli* modification spectrum is very similar to that recently determined for Human HEK293 cells [66] and a large collection of human proteomics data from the PRIDE database [67]. Both of these studies found high counts of +1 Da modfications, ammonia loss (−17 Da), dehydration (−18 Da), and mono- and dioxidation (+16 and +32 Da); in contrast with our results, these studies also identified high levels of carbamylation (+43 Da) and phosphorylation (+80 Da). Whether these differences are due to genuine variability in proteome-wide modification levels or to differences in experimental procedures is unclear.

We have identified examples of abundant modification for N-terminal acetylation and C-terminal glutamylation. While further work is necessary to establish that the orders-of-magnitude differences in abundance between these modifications and more well-known regulatory modifications reflects their actual abundance in the cell, our findings do suggest that these modifications may play a more important physiological role than previously thought. Both of these modifications are known to be installed in a regulated and specific pattern on ribosomal proteins, but their function either in the ribosomal context or on other targets is largely unknown. In eukaryotic cells, N-terminal (Nt) acetylation has a variety of functions, including regulating protein stability, ER trafficking, protein complex formation, and membrane attachment [68], but there is no evidence for a similar role in prokaryotic cells. Nt acetylation of *E. coli* 30S ribosomal subunits S5 and S18 is thought to affect 30S ribosomal assembly by governing direct contacts with the rRNA [69], but no function for prokaryotic Nt acetylation outside of the ribosome has been proposed. While Nt acetylation of eukaryotic proteins can either inhibit [70] or enhance [71] degradation rates, our evidence suggests that Nt-acetylated proteins in *E. coli* are subject to lower levels of degradation than their unmodified counterparts. The viability of mutants in the three known *E. coli* Nt-acetyltransferase enzymes, RimI, RimJ, and RimL [53], should make experimental investigation of this hypothesis a tractable and interesting avenue for future research.

Similarly, the physiological role of C-terminal (Ct) glutamylation has only recently begun to be uncovered. Early investigations identified a Ct glutamyltransferase enzyme, RimK, that installs poly-E tails on ribosomal protein S6 in vivo and in vitro [72], but the only phenotypic effect observed in *E. coli* *rimK* mutant strains (other than loss of S6 glutamylation) is increased resistance to the aminoglycoside antibiotics streptomycin, neomycin, and kanamycin [73, 74]. Nonetheless, RimK, and presumably S6 Ct glutamylation, are conserved across a wide range of bacterial species [75], and recent work in *Pseudomonas* found profound changes in proteome composition and compromised colonization and virulence phenotypes in *Δ* *r* *i* *m* *K* strains [76]. Our novel finding of an additional target of C-terminal glutamylation, the ribosomal hibernation factor YfiA, offers an additional experimental handle with which to examine the biological and molecular functions of this modification. The association of both Ct-glutamylation target proteins with the ribosome is especially interesting, because some evidence suggests that RimK modifies S6 C-termini specifically on intact ribosomes [75, 77], and RimK is known to catalyze poly-L-glutamine formation in the absence of S6 [78]. The C-terminal amino-acid residues of YfiA resemble those of S6 only in the presence of two glutamate residues in the last two positions (DDAEAGDSEE for S6 and ANFVEEVEEE for YfiA), indicating that targeting may largely be a function of YfiA’s structural association with the ribosome rather than due to a specific sequence signal.

The presence of a gradual increase in asparagine deamidation throughout our stationary phase samples is an intriguing observation. Asparagine deamidation/isomerization events occur spontaneously at a low frequency at specific protein residues with favorable local structure and sequence context [61, 63], and they are often observed in proteins that undergo a low frequency of turnover such as muscle fiber proteins [79] and lens crystallins [80]. This clock-like behavior of Asp deamidation is consistent with our observation of a steady accumulation of the Asp +1 Da mark through very late stationary phase (336h), and it suggests that proteins having this modification have been retained with little or no turnover throughout stationary phase. Remarkably, many of the most heavily modified target proteins are part of large supra-molecular complexes, including six on ribosomal proteins (N113 and N544 of ribosomal protein S1, encoded by the *rpsA* gene, N77 and N146 of ribosomal protein S5, encoded by the *rpsE* gene, and N89 of ribosomal protein L14, encoded by the *rplN* gene), N64 of EFTu, N77 of the genomic DNA structural protein H-NS [81], and two positions (N110 and N111) on SucB, the E2 subunit of the 2-oxoglutarate dehydrogenase multienzyme complex (OGDHC) [82]. Although retention of intact ribosomes through stationary phase is a well-documented phenomenon [22, 23], and H-NS has been shown to be involved in late-stationary-phase survival [83], retention of the OGDHC complexes has not been previously observed.

Our work has several limitations. First, although the consistent temporal signal across multiple replicates strongly indicates that the major modifications discussed above are of biological origin, we cannot rule out the possibility that a subset of these modifications are experimental artifacts; the oxidative modifications and asparagine deamidation in particular are known to occur as artifacts of downstream sample processing in MS/MS [65, 84], so further experimental verification will be needed to confirm their biological origin. Future studies applying our approach to datasets generated from PTM-installing enzyme mutant strains would be especially informative, as would applying targeted mass spectrometry methods such as Parallel Reaction Monitoring (PRM) [85] for precise quantitation of modification levels and stoichiometry.

Our study is also limited by the need to examine a relatively small window of mass shifts (−200 Da to +200 Da); many known modifications fall outside of this window, such as glycosylation, longer chain acylations, and lipidations [86]. In addition, while the lack of bias for particular modifications offers a number of advantages in our analysis strategy, it also means that our results are more limited by the inherent sensitivity of both shotgun MS/MS and computational identification of PTMs. Consequently, our data are biased towards highly abundant proteins and mass shifts, a factor that likely explains the scarcity of well-known PTMs such as lysine acetylation and phosphorylation in our data. Equipment improvements and/or novel experimental procedures (e.g. [87]) will likely be needed to enable detection of low-abundance or short-lived PTM and other rare effects such as translational mutations [88].

While our primary focus in this work was on discovering novel PTM biology, we anticipate that our findings will be of value to the mass spectrometry community more generally. Our results highlight the utility of unbiased, mass-shift-aware search strategies for database mapping of spectra containing unexpected PTMs that may otherwise have remained unassigned [67]. Furthermore, our results demonstrate that the presence of unexpected PTM can strongly influence the accuracy of spectral-counting-based estimates of peptide abundance, particularly when the goal is to determine expression patterns over time. For example, we identified a number of proteins where the temporal pattern of the Nt-acetylated peptide differs markedly from the unmodified peptide (see Fig. 14), and from the combined peptide total. If only the unmodified spectra were assigned for a protein such as YadR (Fig. 14, left column, second plot from top), abundance of this peptide would appear to decrease at later timepoints in stationary growth; if both modified and modified peptides are examined, it becomes clear that abundance remains constant across the growth cycle.

## Conclusions

In summary, the work presented here highlights the holistic perspective and novel biological insights that can be generated by combining unbiased PTM detection and deep temporal sampling of bacterial growth. Stationary phase biology and post-translational modification in prokaryotic systems are both still areas of active research with many open questions, and we hope that the analysis paradigm presented here can be applied to additional organisms and growth conditions to gain broader insight into prokaryotic physiology and evolution.

## Methods

### Origin of the analyzed data

All data were taken from a previously published *E. coli* time course [39]. In that study, *E. coli* was grown in glucose minimal media and samples were collected at 8 different time points: 3, 4, 5, 8, 24, 48, 168 and 336 hours past inoculation. The entire experiment was carried out in triplicate, with cultures in each time course grown at different times. Mass-spectrometry on these samples was carried out as follows [39]: Protein samples were prepared by trypsin digest and each sample was then analyzed using liquid chromatography mass spectrometry (LC/MS) on a LTQ-Orbitrap (Thermo Fisher). The resulting data are available from the ProteomeXchange Consortium (accession PXD002140) [89].

### Post-translational modification identification and analysis

We analyzed the raw mass-spectrometry data via MODa [38]. MODa is a naive Bayes spectral alignment algorithm that identifies peptides and their associated PTMs from the input mzXML spectral files. The program needs a few additional parameters, such as enzyme used, instrument used to capture the mass-spec data, precursor and product ion mass tolerances, fixed modifications, any rules to apply on the digest, such as semi-tryptic or fully-tryptic, number of modifications per peptide, and the mass-range to search for PTMs. We ran separate MODa searches for each of the 9 time points. Since there were 3 biological replicates, this resulted in a total of 27 MODa searches. We set the enzyme used in the searches to trypsin, with fully-tryptic and no-proline rules. We allowed for 2 missed cleavages. We used a mass-tolerance for the precursor ion of 10 ppm, and the mass-tolerance used for the product ion was set to 0.5 Da. Finally, we set carbamidomethylation (+57 Da) of cysteine as a static or fixed modification. As mentioned earlier, MODa requires a mass range to search for variable modifications, so we ran MODa searches for the mass range between −200 and +200 Da. We used the *E. coli* B REL606 genome sequence (GenBank:NC_012967.1 [90]) to create the reference proteome.

### FDR calculations using MODa probabilities

For each PSM assigned to a spectrum by MODa, the algorithm calculates a probability *P*_MODa_ using a logistic regression model that uses a variety of spectral features as parameters, trained on a standard set of correct and incorrect spectral matches [38]. To restrict our dataset to only high-quality PSMs, we used this probability to estimate the False-Discovery Rate (FDR) of incorrect matches in our dataset by (i) ranking all PSMs by their *P*_MODa_ values, and then (ii) iteratively adding PSMs, starting from the highest-probability matches, and calculating the FDR as

$$\text{FDR} = \frac{1}{k}\sum_{i=1}^{k} (1 - P_{i}), $$

where *k* is the rank index of the last added PSM and *P*_*i*_ is the *P*_MODa_ of the *i*th ranked PSM, until adding any additional PSMs would result in an FDR above the chosen cutoff value.

### Metrics and statistical tests for single amino-acid bias, N-terminal/C-terminal bias, and growth-phase bias

To test the preference of each mass shift for modification of a single type of amino acid, we calculated a single-AA bias score *B*_*s*_(*A*) for mass shift *s* and length 20 vector *A* of counts of unique positions bearing at least one modification matching *s* for each amino acid type:

$$B_{s}(A) = \frac{U_{m}(A) + 1}{\bar{U}_{nm}(A) + 1} $$

where *U*_*m*_(*A*)= max(*U*_*a*∈*A*_) and $\bar {U}_{nm}(A) = \frac {1}{19}\sum _{a \ne m}U_{a}$. Note that “unique position” means that a given position in a protein is counted at most once regardless of total PSM counts at that position; this choice was intended to reduce bias from modifications with high abundance at a small number of positions.

To simplify our analysis, we constructed an intermediate dataset of PSM counts calculated by amino-acid position across all proteins in the REL606 annotated proteome. Unmodified counts *n*_*p*,unmod_ for each position *p* (having at least one modified or unmodified PSM) were calculated by summing PSM counts for any peptides that overlap *p* but do not have a modification (of any mass shift) at *p*. Modified counts *n*_*p*,*s*_ were calculated by summing PSM counts for any peptides with a modification of mass shift *s* at protein position *p*.

To test for higher fractional modification by specific mass shifts at the protein termini, we constructed 2 × 2 contingency tables of the form shown in Table 9 for each mass shift *s* in each of the three biological replicates, where *N*_Xt_(*s*) is the sum $\sum _{p = \text {Xt}}(n_{p, s} + {n_{p, \text {unmod}}})$ for positions having at least one PSM with mass shift *s* occuring at the terminus Xt (either C- or N-terminus of a protein); *N*_non-Xt_(*s*) is the sum $\sum _{p \ne \text {Xt}}(n_{p, s} + {n_{p, \text {unmod}}})$ for positions *p* having at least one PSM with mass shift *s*, occuring at all other positions (including the opposite terminus); *N*_mod_(*s*) is the sum $\sum {n_{p, s}}$ for positions having at least one PSM with mass shift *s*; and *N*_unmod_(*s*) is the sum $\sum {n_{p, \text {unmod}}}$ for positions having at least one PSM with mass shift *s*. We used these tables to perform Fisher’s exact tests using a two-sided alternative hypothesis, implemented in Python using the statistics module of NumPy[91].

Similarly, to test for higher fractional modification by specific mass shift × amino acid pairs in either exponential or stationary phases of growth, we constructed 2×2 contingency tables of the form shown in Table 10 for each mass-shift–amino-acid pair in the three biological replicates, where *N*_mod_(*s*,*a*) and *N*_unmod_(*s*,*a*) are as above and *N*_EXP_(*s*,*a*) is the sum $\sum _{t=3, 4, 5, 6}(n_{p, s, t} + {n_{p, \text {unmod}, t}})$, where *n*_*p*,*s*,*t*_ is the count of PSMs modified by mass shift *s* at position *p* in timepoint *t* for positions *p* of amino acid type *a* having at least one PSM with mass shift *s*; and *N*_STA_(*s*,*a*) is the sum $\sum _{t=24, 48, 168, 336}(n_{p,s,t} + {n_{p, \text {unmod}, t}})$ for positions *p* of amino acid type *a* having at least one PSM with mass shift *s*. We used these tables to perform Fisher’s exact tests using a two-sided alternative hypothesis, implemented in Python using the statistics module of NumPy [91].

**Table 9:** 2×2 contingency table for Fisher’s Exact Test for N-terminal- and C-terminal-enriched mass shifts

	*N*_mod_(*s*)	*N*_unmod_(*s*)
*N*_Xt_(*s*)	*N*_Xt, mod_(*s*)	*N*_Xt, unmod_(*s*)
*N*_non−Xt_(*s*)	*N*_non−Xt, mod_(*s*)	*N*_non−Xt, unmod_(*s*)

**Table 10:** 2×2 contingency table for the Fisher’s Exact Test for exponential- or stationary-phase enriched mass shifts

	*N*_mod_(*s*,*a*)	*N*_unmod_(*s*,*a*)
*N*_STA_(*s*,*a*)	*N*_STA, mod_(*s*,*a*)	*N*_STA, unmod_(*s*,*a*)
*N*_EXP_(*s*,*a*)	*N*_EXP, mod_(*s*,*a*)	*N*_EXP, unmod_(*s*,*a*)

Analysis of sequence composition for +1 Da modifications was performed by first splitting all +1 Da modified positions into modifications localized by MODa to asparagine residues and modifications localized to non-asparagine residues. Each of these groups was ranked by total PSM counts, and a +/− 5 amino acid sequence window centered at the modified residue was extracted for the top 50 modified positions in each group. Sequences were then submitted to WebLogo [92] to construct sequence logos using default settings.

### Additional software used for analysis

The analysis was performed in iPython [93] notebooks using the NumPy and SciPy libraries [91] for numerical calculations, the Pandas library [94] for data processing, and the MatPlotLib library [95] for plotting. Macromolecular structures in Additional file 4 were assembled in MacPyMOL (version v1.7.4.4; Schrödinger, LLC).

## Additional files

Additional file 1 Abundance of all observed mass shifts across all 9 timepoints and 3 biological replicates. Color of heatmap corresponds to the log2-transformed count of MODa-called modified PSMs in the 1% FDR set bearing the mass shift indicated on the *y*-axis for each of the nine timepoints (*x*-axis), for biological replicates 1, 2, and 3 (left, center, and right panels respectively). Although the MODa analysis was conducted for the mass window from −200 to +200 Da, no modifications were identified with mass shifts below −130 Da or above +196 Da. (PNG 258 kb)

Additional file 2 Zip file containing several data tables in tab-separated format, as well as a readme file that explains the contents of each data file. (ZIP 175 kb)

Additional file 3 Temporally variable modification for individual proteins with an N-terminal serine possessing a +42 Da modification. The plots show unmodified (green) and +42 Da Modified (blue) PSM counts across all nine timepoints (*x*-axis) for the N-terminal position of all proteins that have both (i) at least one PSM identified by MODa as containing an N-terminal +42 Da modification and (ii) having a penultimate serine (AA position 2; i.e. the N-terminal residue following N-terminal methionine cleavage). Counts represent the average of the three biological replicates. Plots are ordered from top to bottom by the mean *p* value of the Fisher’s exact test for preferential modification (see text) from left-to-right within each row, and top-to-bottom across rows, with the most significant protein at the top left. (PDF 24 kb)

Additional file 4 Relative locations of YfiA (blue) and native *T. thermophilus* S6 (magenta) proteins in crystal structure of *E. coli* YfiA bound to the *T. thermophilus* 70S ribosome (PDB ID 4V8I [49]). YfiA is positioned within the 30S subunit mRNA tunnel, and S6 on the outer surface of the 30S subunit; the C-terminal tails of both proteins (black arrows) point toward the same region of the 16S rRNA (light blue). The 17 C-terminal residues for YfiA, including the terminal glutamate residues, were not resolved in the crystal structure; the *T. thermophilus* S6 protein coding sequence ends at residue 101, lacking the 30-AA unstructured C-terminal domain present in *E. coli* S6. 16S rRNA is shown in light blue; 30S ribosomal proteins (other than S6) are shown in light yellow; 50S ribosomal proteins are shown in green; and 23S rRNA is shown in pink. (PNG 3630 kb)

Additional file 5 Modified and unmodified PSM counts for each AA position with a C-terminal +129 Da modification across all timepoints. The plots show unmodified (green) and +129 Da modified (purple) PSM counts across all nine timepoints (*x*-axis) for the C-terminal position of the two proteins that have at least one PSM identified by MODa as containing a C-terminal +129 Da modification. Counts represent the average of the three biologcial replicates. (PDF 14 kb)

Additional file 6 Temporally variable modification for individual proteins with an N-terminal threonine possessing a +42 Da modification. The plots show unmodified (green) and +42 Da Modified (blue) PSM counts across all nine timepoints (*x*-axis) for the N-terminal position of all proteins that have both (i) at least one PSM identified by MODa as containing an N-terminal +42 Da modification and (ii) having a penultimate threonine (AA position 2; i.e. the N-terminal residue following N-terminal methionine cleavage). Counts represent the average of the three biological replicates. Plots are ordered from top to bottom by the mean *p* value of the Fisher’s exact test for preferential modification (see text) from top to bottom, with the most significant protein at the top. (PDF 17 kb)

Additional file 7 Fraction of total peptides across timepoints with an N-terminal alanine possessing a +42 Da modification. The plots show unmodified (green) and +42 Da modified (blue) PSM counts across all nine timepoints (*x*-axis) for the N-terminal position of all proteins that have both (i) at least one PSM identified by MODa as containing an N-terminal +42 Da modification and (ii) having a penultimate Alanine (AA position 2; i.e. the N-terminal residue following N-terminal methionine cleavage). Counts represent the average of the three biologcial replicates. Plots are ordered from top to bottom by the mean *p* value of the Fisher’s exact test for preferential modification (see text) from top to bottom, with the most significant protein at the top. (PDF 17 kb)

Additional file 8 Modified and unmodified PSM counts for each AA position with a significantly stationary-phase biased +1 Da modification to asparagine. The plots show unmodified (green) and +1 Da modified (brown) PSM counts across all nine timepoints (*x*-axis) for the 10 asparagine residues with the most significant *p*-values across all three biological replicates. Counts represent the average of the three biologcial replicates. Plots are ordered by the mean *p* value of the Fisher’s exact test for preferential modification from left-to-right within each row, and from top-to-bottom across rows, with the most significant position at the top left. (PDF 20 kb)

Additional file 9 Amino Acid sequence logos generated using WebLogo [92] for a +/- 5 AA window around the MODa-called site of modification for the top 50 most abundant +1Da modifications localized at Asparagine residues (A) and at all other residue types combined (B). Asparagine residues show a preferential enrichment of Glycine, Serine, and Asparagine AAs at the +1 position not observed for non-Asn modifications. (PDF 347 kb)

Additional file 10 Modified and unmodified counts across timepoints for all AA positions with a +16 Da modification to methionine, pooled by biological replicate. The plots show the total unmodified (green) and +16 Da modified (magenta) PSM counts across all nine timepoints (*x*-axis) for methionine residues that have at least one +16 Da modification at any time point in any replicate. The three panels show counts for each of the three biological replicates, replicate 1 (A), replicate 2 (B) and replicate 3 (C). Note that the *y*-axis is plotted on a logarithmic (base 10) scale due to the high number of total counts relative to modified counts. (PDF 16 kb)

Additional file 11 Modified and unmodified counts across timepoints for all AA positions with a +16 Da modification to tryptophan, pooled by biological replicate. The plots show the total unmodified (green) and +16 Da modified (orange) PSM counts across all nine timepoints (*x*-axis) for tryptophan residues that have at least one +16 Da modification at any time point in any replicate. The three panels show counts for each of the three biological replicates 1 (A), 2 (B) and 3 (C). Note that the *y*-axis is plotted on a logarithmic (base 10) scale due to the high number of total counts relative to modified counts. (PDF 16 kb)

Additional file 12 Modified and unmodified counts across timepoints for the top 10 exponential-enriched AA positions with a +16 Da modification to methionine. The plots show unmodified (green) and +16 Da modified (magenta) methionine PSM counts across all nine timepoints (*x*-axis) for the protein and position indicated. Plots in columns correspond to the three biological replicates 1 (left column), 2 (center column), and 3 (right column). Counts represent the average of the three biologcial replicates. Plots are ordered from top to bottom by the mean *p* value of the Fisher’s exact test for preferential modification (see text), with the most significant protein at the top. (PDF 25 kb)

Additional file 13 Modified and unmodified counts across timepoints for the top 15 exponential-enriched AA positions with a +16 Da modification to tryptophan. The plots show unmodified (green) and +16 Da modified (orange) tryptophan PSM counts across all nine timepoints (*x*-axis) for the protein and position indicated. Plots in columns correspond to the three biological replicates 1 (left column), 2 (center column), and 3 (right column). Counts represent the average of the three biologcial replicates. Plots are ordered from top to bottom by the mean *p* value of the Fisher’s exact test for preferential modification (see text), with the most significant protein at the top. (PDF 25 kb)

## Abbreviations

AA: Amino acid
Ct: C-terminal
EXP: Exponential phase
FDR: False-discovery rate
FET: Fisherʼs exact test
GASP: Growth advantage in stationary phase
IAA: Iodoacetamide
Nt: N-terminal
NtAc: N-terminal acetylation
OGDHC: 2-oxyglutarate dehydrogenase multienzyme complex
PTM: Post-translational modification
PSM: Peptide spectral match
STA: Stationary phase
VBNC: Viable but non-culturable

## References

[1] Khoury GA, Baliban RC, Floudas CA. Proteome-wide post-translational modification statistics: frequency analysis and curation of the swiss-prot database. Sci Rep. 2011;1. doi:10.1038/srep00090. 10.1038/srep00090 PMC3201773 22034591

[2] Jenuwein T, Allis CD (2001). Translating the histone code. Science.

[3] Pawson T (1995). Protein modules and signalling networks. Nature.

[4] Lim WA (2002). The modular logic of signaling proteins: building allosteric switches from simple binding domains. Curr Opin Struct Biol.

[5] Laub MT, Goulian M (2007). Specificity in two-component signal transduction pathways. Annu Rev Genet.

[6] Choudhary C, Mann M (2010). Decoding signalling networks by mass spectrometry-based proteomics. Nat Rev Mol Cell Biol.

[7] Jones JD, O’Connor CD (2011). Protein acetylation in prokaryotes. Proteomics.

[8] Dworkin J (2015). Ser/Thr phosphorylation as a regulatory mechanism in bacteria. Curr Opin Microbiol.

[9] Kusebauch U, Ortega C, Ollodart A, Rogers RS, Sherman DR, Moritz RL, Grundner C (2014). Mycobacterium tuberculosis supports protein tyrosine phosphorylation. Proc Natl Acad Sci U S A.

[10] Hansen A-MM, Chaerkady R, Sharma J, Díaz-Mejía JJ, Tyagi N, Renuse S, Jacob HKC, Pinto SM, Sahasrabuddhe NA, Kim M-SS, Delanghe B, Srinivasan N, Emili A, Kaper JB, Pandey A (2013). The *Escherichia coli* phosphotyrosine proteome relates to core pathways and virulence. PLoS Pathog.

[11] Striebel F, Imkamp F, Özcelik D, Weber-Ban E (2014). Pupylation as a signal for proteasomal degradation in bacteria. Biochim Biophys Acta.

[12] Alber T (2009). Signaling mechanisms of the mycobacterium tuberculosis receptor Ser/Thr protein kinases. Curr Opin Struct Biol.

[13] Ouidir T, Jarnier F, Cosette P, Jouenne T, Hardouin J. Characterization of N-terminal protein modifications in *Pseudomonas aeruginosa* PA14,. J Proteomics. 2014. doi:10.1016/j.jprot.2014.11.006. 10.1016/j.jprot.2014.11.006 25464366

[14] Hentchel KL, Escalante-Semerena JC (2015). Acylation of biomolecules in prokaryotes: a widespread strategy for the control of biological function and metabolic stress. Microbiol Mol Biol Rev.

[15] Starai VJ, Escalante-Semerena JC (2004). Identification of the protein acetyltransferase (Pat) enzyme that acetylates acetyl-CoA synthetase in *Salmonella enterica*. J Mol Biol.

[16] Salomon D, Orth K (2013). What pathogens have taught us about posttranslational modifications. Cell Host Microbe.

[17] Gnad F, Forner F, Zielinska DF, Birney E, Gunawardena J, Mann M (2010). Evolutionary constraints of phosphorylation in eukaryotes, prokaryotes, and mitochondria. Mol Cell Proteomics.

[18] Navarro Llorens JM, Tormo A, Martínez-García E (2010). Stationary phase in gram-negative bacteria. FEMS Microbiol Rev.

[19] Finkel SE (2006). Long-term survival during stationary phase: evolution and the GASP phenotype. Nat Rev Microbiol.

[20] Nyström T (1994). The glucose-starvation stimulon of *Escherichia coli*: induced and repressed synthesis of enzymes of central metabolic pathways and role of acetyl phosphate in gene expression and starvation survival. Mol Microbiol.

[21] Reeve CA, Amy PS, Matin A (1984). Role of protein synthesis in the survival of carbon-starved *Escherichia coli* K-12. J Bacteriol.

[22] Maki Y, Yoshida H, Wada A (2000). Two proteins, YfiA and YhbH, associated with resting ribosomes in stationary phase *Escherichia coli*,. Genes Cells.

[23] Wada A (1998). Growth phase coupled modulation of *Escherichia coli* ribosomes. Genes Cells.

[24] Nyström T (2002). Translational fidelity, protein oxidation, and senescence: lessons from bacteria. Ageing Res Rev.

[25] Petropoulos I, Friguet B (2005). Protein maintenance in aging and replicative senescence: a role for the peptide methionine sulfoxide reductases. Biochim Biophys Acta.

[26] Weichart D, Querfurth N, Dreger M, Hengge-Aronis R (2003). Global role for ClpP-containing proteases in stationary-phase adaptation of *Escherichia coli*. J Bacteriol.

[27] Na SH, Miyanaga K, Unno H, Tanji Y (2006). The survival response of *Escherichia coli* K12 in a natural environment. Appl Microbiol Biotechnol.

[28] Schmidt A, Kochanowski K, Vedelaar S, Ahrné E, Volkmer B, Callipo L, Knoops K, Bauer M, Aebersold R, Heinemann M (2016). The quantitative and condition-dependent *Escherichia coli* proteome. Nat Biotechnol.

[29] Soufi B, Krug K, Harst A, Macek B (2015). Characterization of the *E. coli* proteome and its modifications during growth and ethanol stress. Front Microbiol.

[30] Weinert BT, Iesmantavicius V, Wagner SA, Schölz C, Gummesson B, Beli P, Nyström T, Choudhary C (2013). Acetyl-phosphate is a critical determinant of lysine acetylation in *E. coli*. Mol Cell.

[31] Soares NC, Spät P, Krug K, Macek B (2013). Global dynamics of the *Escherichia coli* proteome and phosphoproteome during growth in minimal medium. J Proteome Res.

[32] Kuhn ML, Zemaitaitis B, Hu LI, Sahu A, Sorensen D, Minasov G, Lima BP, Scholle M, Mrksich M, Anderson WF, Gibson BW, Schilling B, Wolfe AJ (2014). Structural, kinetic and proteomic characterization of acetyl phosphate-dependent bacterial protein acetylation. PLoS ONE.

[33] Macek B, Mann M, Olsen JV (2009). Global and site-specific quantitative phosphoproteomics: principles and applications. Annu Rev Pharmacol Toxicol.

[34] Perkins DN, Pappin DJ, Creasy DM, Cottrell JS (1999). Probability-based protein identification by searching sequence databases using mass spectrometry data. Electrophoresis.

[35] Eng JK, McCormack AL, Yates JR (1994). An approach to correlate tandem mass spectral data of peptides with amino acid sequences in a protein database. J Am Soc Mass Spectrom.

[36] Geer LY, Markey SP, Kowalak JA, Wagner L, Xu M, Maynard DM, Yang X, Shi W, Bryant SH (2004). Open mass spectrometry search algorithm. J Proteome Res.

[37] Craig R, Beavis RC (2004). TANDEM: matching proteins with tandem mass spectra. Bioinformatics.

[38] Na S, Bandeira N, Paek E (2012). Fast multi-blind modification search through tandem mass spectrometry. Mol Cell Proteomics.

[39] Houser JR, Barnhart C, Boutz DR, Carroll SM, Dasgupta A, Michener JK, Needham BD, Papoulas O, Sridhara V, Sydykova DK, Marx CJ, Trent MS, Barrick JE, Marcotte EM, Wilke CO (2015). Controlled measurement and comparative analysis of cellular components in *E. coli* reveals broad regulatory changes in response to glucose starvation. PLoS Comput Biol.

[40] Chalkley RJ, Baker PR, Medzihradszky KF, Lynn AJ, Burlingame AL (2008). In-depth analysis of tandem mass spectrometry data from disparate instrument types. Mol Cell Proteomics.

[41] Wiśniewski JR, Rakus D (2014). Quantitative analysis of the *Escherichia coli* proteome. Data Brief.

[42] Fu Y, Qian X (2014). Transferred subgroup false discovery rate for rare post-translational modifications detected by mass spectrometry. Mol Cell Proteomics.

[43] Hart-Smith G, Yagoub D, Tay AP, Pickford R, Wilkins MR (2016). Large scale mass spectrometry-based identifications of enzyme-mediated protein methylation are subject to high false discovery rates. Mol Cell Proteomics.

[44] Zhang K, Zheng S, Yang JS, Chen Y, Cheng Z (2013). Comprehensive profiling of protein lysine acetylation in *Escherichia coli*,. J Proteome Res.

[45] SPAHR PF (1962). Amino acid composition of ribosomes from *Escherichia coli*. J Mol Biol.

[46] Scherl A, Shaffer SA, Taylor GK, Hernandez P, Appel RD, Binz P. -AA, Goodlett DR (2008). On the benefits of acquiring peptide fragment ions at high measured mass accuracy. J Am Soc Mass Spectrom.

[47] Reeh S, Pedersen S (1979). Post-translational modification of *Escherichia coli* ribosomal protein S6. Mol Gen Genet.

[48] Vila-Sanjurjo A, Schuwirth B. -SS, Hau CW, Cate JHD (2004). Structural basis for the control of translation initiation during stress. Nat Struct Mol Biol.

[49] Polikanov YS, Blaha GM, Steitz TA (2012). How hibernation factors RMF, HPF, and YfiA turn off protein synthesis. Science.

[50] Agafonov DE, Kolb VA, Spirin AS (2001). Ribosome-associated protein that inhibits translation at the aminoacyl-tRNA binding stage. EMBO Rep.

[51] Ueta M, Yoshida H, Wada C, Baba T, Mori H, Wada A (2005). Ribosome binding proteins YhbH and YfiA have opposite functions during 100S formation in the stationary phase of *Escherichia coli*. Genes Cells.

[52] Fisher RA (1922). On the interpretation of *χ* 2 from contingency tables, and the calculation of p. J R Stat Soc.

[53] Nesterchuk MV, Sergiev PV, Dontsova OA (2011). Posttranslational modifications of ribosomal proteins in *Escherichia coli*,. Acta Naturae.

[54] Arai K, Clark BF, Duffy L, Jones MD, Kaziro Y, Laursen RA, L’Italien J, Miller DL, Nagarkatti S, Nakamura S, Nielsen KM, Petersen TE, Takahashi K, Wade M (1980). Primary structure of elongation factor Tu from *Escherichia coli*. Proc Natl Acad Sci U S A.

[55] Smith VF, Schwartz BL, Randall LL, Smith RD (1996). Electrospray mass spectrometric investigation of the chaperone SecB. Protein Sci.

[56] Bernal-Perez LF, Sahyouni F, Prokai L, Ryu Y (2012). RimJ-mediated context-dependent N-terminal acetylation of the recombinant Z-domain protein in *Escherichia coli*. Mol Biosyst.

[57] Miao L, Fang H, Li Y, Chen H (2007). Studies of the in vitro Nalpha-acetyltransferase activities of *E. coli* RimL protein. Biochem Biophys Res Commun.

[58] Wu J, Chang S, Gong X, Liu D, Ma Q (2006). Identification of N-terminal acetylation of recombinant human prothymosin alpha in *Escherichia coli*. Biochim Biophys Acta.

[59] Charbaut E, Redeker V, Rossier J, Sobel A (2002). N-terminal acetylation of ectopic recombinant proteins in *Escherichia coli*. FEBS Lett.

[60] Lindner H, Helliger W (2001). Age-dependent deamidation of asparagine residues in proteins. Exp Gerontol.

[61] Robinson NE, Robinson AB (2001). Molecular clocks. Proc Natl Acad Sci U S A.

[62] Stephenson RC, Clarke S (1989). Succinimide formation from aspartyl and asparaginyl peptides as a model for the spontaneous degradation of proteins. J Biol Chem.

[63] Robinson NE, Robinson ZW, Robinson BR, Robinson AL, Robinson JA, Robinson ML, Robinson AB (2004). Structure-dependent nonenzymatic deamidation of glutaminyl and asparaginyl pentapeptides. J Pept Res.

[64] Hao P, Ren Y, Alpert AJ, Sze SK (2011). Detection, evaluation and minimization of nonenzymatic deamidation in proteomic sample preparation. Mol Cell Proteomic.

[65] Ghesquière B, Gevaert K (2014). Proteomics methods to study methionine oxidation. Mass Spectrom Rev.

[66] Chick JM, Kolippakkam D, Nusinow DP, Zhai B, Rad R, Huttlin EL, Gygi SP (2015). A mass-tolerant database search identifies a large proportion of unassigned spectra in shotgun proteomics as modified peptides. Nat Biotechnol.

[67] Griss J, Perez-Riverol Y, Lewis S, Tabb DL, Dianes JA, Del-Toro N, Rurik M, Walzer MW, Kohlbacher O, Hermjakob H, Wang R, Vizcaíno JA (2016). Recognizing millions of consistently unidentified spectra across hundreds of shotgun proteomics datasets. Nat Methods.

[68] Starheim KK, Gevaert K, Arnesen T (2012). Protein N-terminal acetyltransferases: when the start matters. Trends Biochem Sci.

[69] Clatterbuck Soper SF, Dator RP, Limbach PA, Woodson SA (2013). In vivo X-ray footprinting of pre-30S ribosomes reveals chaperone-dependent remodeling of late assembly intermediates. Mol Cell.

[70] Martinez A, Traverso JA, Valot B, Ferro M, Espagne C, Ephritikhine G, Zivy M, Giglione C, Meinnel T (2008). Extent of N-terminal modifications in cytosolic proteins from eukaryotes. Proteomics.

[71] Hwang C-SS, Shemorry A, Varshavsky A (2010). N-terminal acetylation of cellular proteins creates specific degradation signals. Science.

[72] Kang WK, Icho T, Isono S, Kitakawa M, Isono K (1989). Characterization of the gene rimK responsible for the addition of glutamic acid residues to the C-terminus of ribosomal protein S6 in *Escherichia coli* K12. Mol Gen Genet.

[73] Kade B, Dabbs ER, Wittmann-Liebold B (1980). Protein-chemical studies on *Escherichia coli* mutants with altered ribosomal proteins S6 and S7. FEBS Lett.

[74] Brown ME, Apirion D (1974). Mapping a cluster of ribosomal genes in *Escherichia coli*. Mol Gen Genet.

[75] Koonin EV, Bork P, Sander C (1994). A novel RNA-binding motif in omnipotent suppressors of translation termination, ribosomal proteins and a ribosome modification enzyme?. Nucleic Acids Res.

[76] Little RH, Grenga L, Saalbach G, Howat AM, Pfeilmeier S, Trampari E, Malone JG (2016). Adaptive remodeling of the bacterial proteome by specific ribosomal modification regulates pseudomonas infection and niche colonisation. PLoS Genet.

[77] Kitakawa M, Blumenthal L, Isono K (1980). Isolation and characterization of specialized transducing lambda phages carrying ribosomal protein genes of *Escherichia coli*. Mol Gen Genet.

[78] Kino K, Arai T, Arimura Y (2011). Poly-alpha-glutamic acid synthesis using a novel catalytic activity of RimK from *Escherichia coli* K-12. Appl Environ Microbiol.

[79] Rivers J, McDonald L, Edwards IJ, Beynon RJ (2008). Asparagine deamidation and the role of higher order protein structure. J Proteome Res.

[80] Hains PG, Truscott RJW (2010). Age-dependent deamidation of lifelong proteins in the human lens. Invest Ophthalmol Vis Sci.

[81] Rimsky S, Travers A (2011). Pervasive regulation of nucleoid structure and function by nucleoid-associated proteins. Curr Opin Microbiol.

[82] Murphy GE, Jensen GJ (2005). Electron cryotomography of the *E. coli* pyruvate and 2-oxoglutarate dehydrogenase complexes. Structure.

[83] Chib S, Mahadevan S (2012). Involvement of the global regulator H-NS in the survival of *Escherichia coli* in stationary phase. J Bacteriol.

[84] Yang H, Zubarev RA (2010). Mass spectrometric analysis of asparagine deamidation and aspartate isomerization in polypeptides. Electrophoresis.

[85] Yang Z, Li N (2015). Absolute quantitation of protein posttranslational modification isoform. Methods Mol Biol.

[86] Walsh CT, Garneau-Tsodikova S, Gatto GJ (2005). Protein posttranslational modifications: the chemistry of proteome diversifications. Angew Chem Int Ed Engl.

[87] Baeza J, Dowell JA, Smallegan MJ, Fan J, Amador-Noguez D, Khan Z, Denu JM (2014). Stoichiometry of site-specific lysine acetylation in an entire proteome. J Biol Chem.

[88] Ribas de Pouplana L, Santos MAS, Zhu J. -H. H., Farabaugh PJ, Javid B (2014). Protein mistranslation: friend or foe?. Trends Biochem Sci.

[89] Vizcaíno JA, Deutsch EW, Wang R, Csordas A, Reisinger F, Ríos D, Dianes JA, Sun Z, Farrah T, Bandeira N, Binz P-AA, Xenarios I, Eisenacher M, Mayer G, Gatto L, Campos A, Chalkley RJ, Kraus H-JJ, Albar JP, Martinez-Bartolomé S, Apweiler R, Omenn GS, Martens L, Jones AR, Hermjakob H (2014). ProteomeXchange provides globally coordinated proteomics data submission and dissemination. Nat Biotechnol.

[90] Jeong H, Barbe V, Lee CH, Vallenet D, Yu DS, Choi S-HH, Couloux A, Lee S-WW, Yoon SH, Cattolico L, Hur C-GG, Park H-SS, Ségurens B, Kim SC, Oh TK, Lenski RE, Studier FW, Daegelen P, Kim JF (2009). Genome sequences of *Escherichia coli* B strains REL606 and BL21(DE3). J Mol Biol.

[91] Van Der Walt S, Colbert SC, Varoquaux G (2011). The NumPy array: a structure for efficient numerical computation. Comput Sci Eng.

[92] Crooks GE, Hon G, Chandonia JM, Brenner SE (2004). WebLogo: a sequence logo generator. Genome Res.

[93] Pérez F, Granger BE (2007). IPython: a system for interactive scientific computing. Comput Sci Eng.

[94] McKinney W (2010). Data structures for statistical computing in python. Proceedings of the 9th Python in Science Conference.

[95] Hunter JD (2007). Matplotlib: A 2d graphics environment. Comput Sci Eng.

[96] Terhorst C, Möller W, Laursen R, Wittmann-Liebold B (1973). The primary structure of an acidic protein from 50-S ribosomes of *Escherichia coli* which is involved in GTP hydrolysis dependent on elongation factors G and T. Eur J Biochem.

[97] Young CC, Bernlohr RW (1991). Elongation factor Tu is methylated in response to nutrient deprivation in *Escherichia coli*. J Bacteriol.

[98] Zhang Z, Tan M, Xie Z, Dai L, Chen Y, Zhao Y (2011). Identification of lysine succinylation as a new post-translational modification. Nat Chem Biol.

[99] Dognin MJ, Wittmann-Liebold B (1980). Purification and primary structure determination of the N-terminal blocked protein, L11, from *Escherichia coli* ribosomes. Eur J Biochem.

