# Supplementary figures and images for: Large-scale analysis of post-translational modifications in E. coli under glucose-limiting conditions
Source: BMC Genomics. 2017 Apr 17;18:301. doi: 10.1186/s12864-017-3676-8 (PMC5392934; doi:10.1186/s12864-017-3676-8)

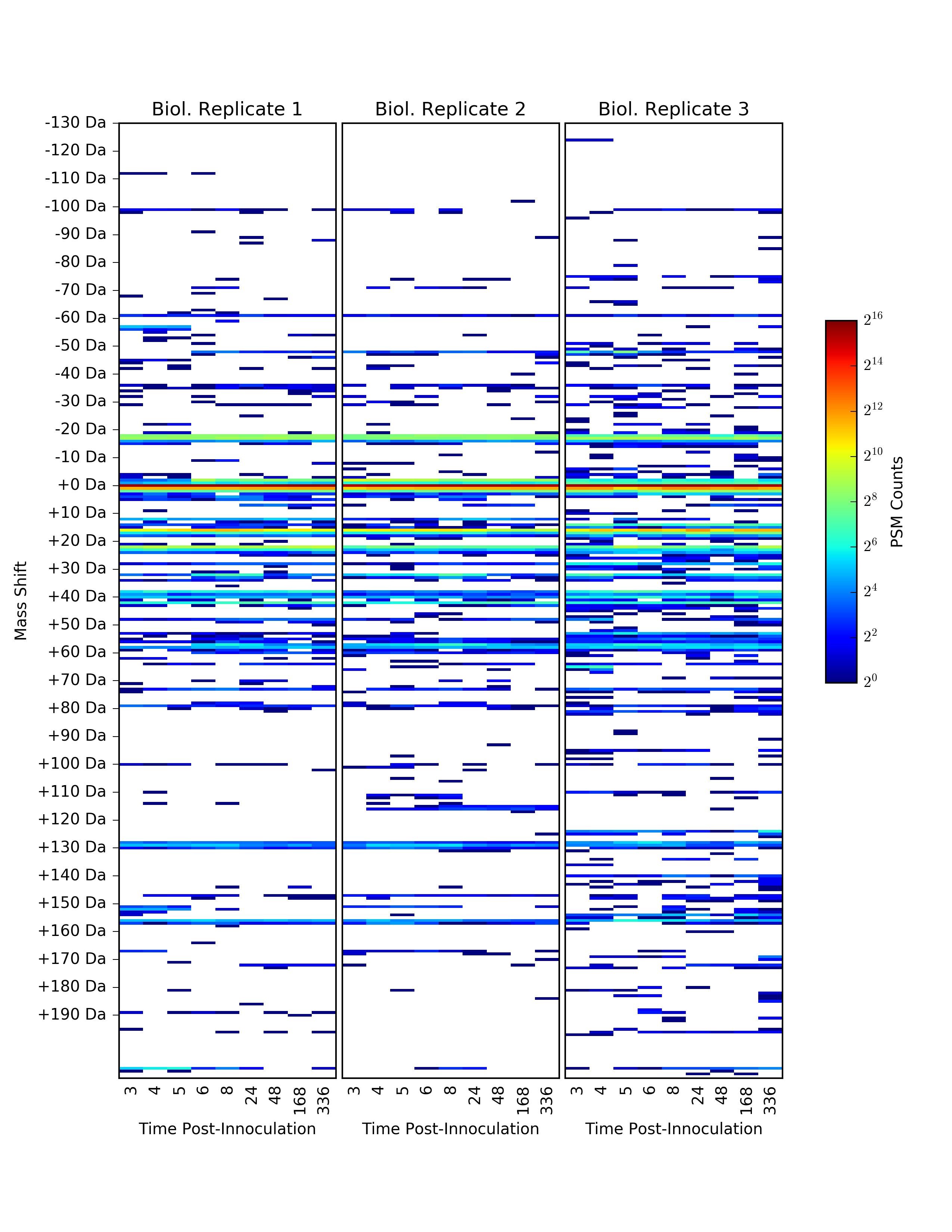

Supplement: Supplementary file 1 — Abundance of all observed mass shifts across all 9 timepoints and 3 biological replicates. Color of heatmap corresponds to the log2-transformed count of MODa-called modified PSMs in the 1% FDR set bearing the mass shift indicated on the y-axis for each of the nine timepoints (x-axis), for biological replicates 1, 2, and 3 (left, center, and right panels respectively). Although the MODa analysis was conducted for the mass window from −200 to +200 Da, no modifications were identified with mass shifts below −130 Da or above +196 Da. (PNG 258 kb) [file 12864_2017_3676_MOESM1_ESM.png]

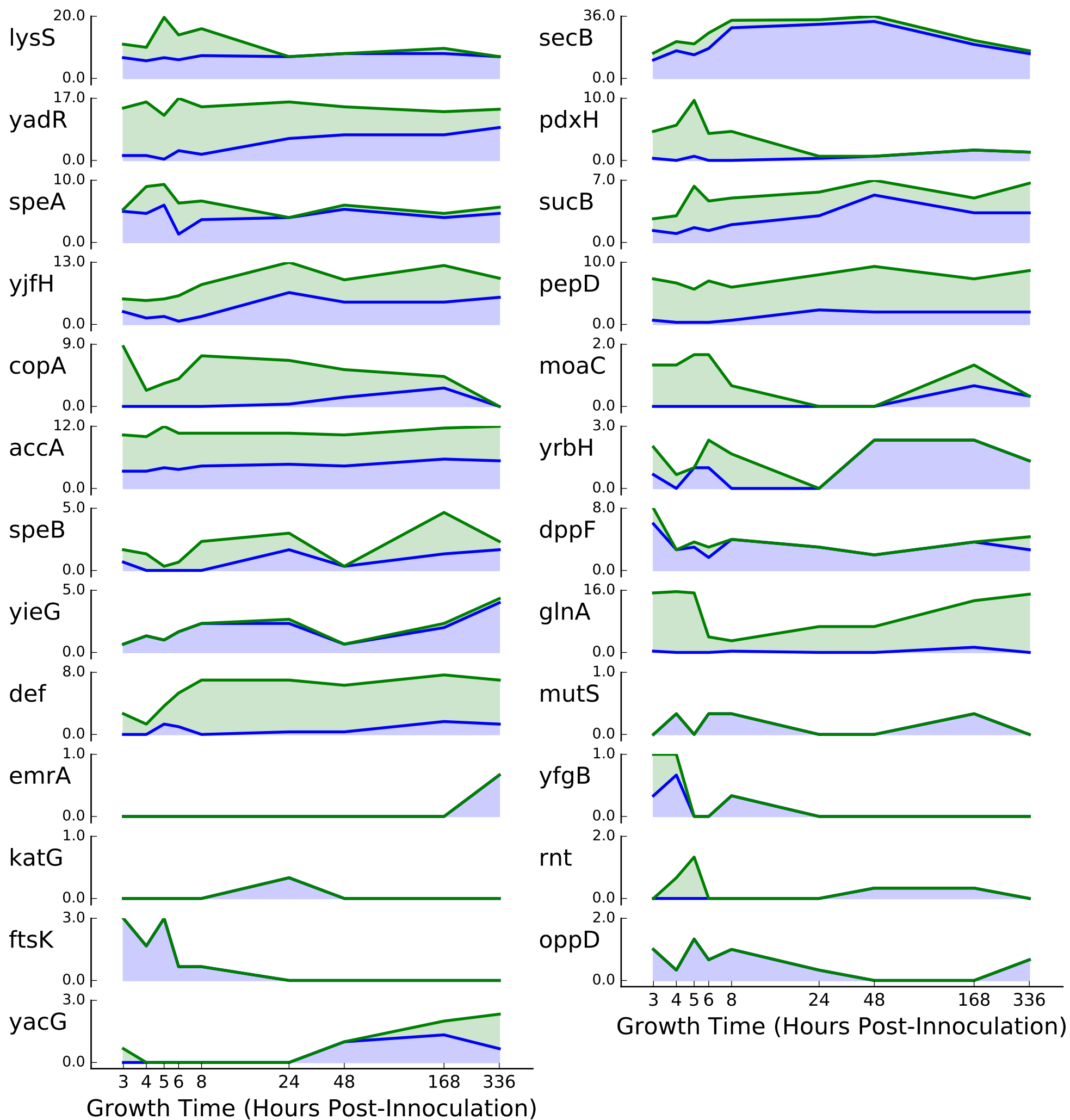

Supplement: Supplementary file 3 — Temporally variable modification for individual proteins with an N-terminal serine possessing a +42 Da modification. The plots show unmodified (green) and +42 Da Modified (blue) PSM counts across all nine timepoints (x-axis) for the N-terminal position of all proteins that have both (i) at least one PSM identified by MODa as containing an N-terminal +42 Da modification and (ii) having a penultimate serine (AA position 2; i.e. the N-terminal residue following N-terminal methionine cleavage). Counts represent the average of the three biological replicates. Plots are ordered from top to bottom by the mean p value of the Fisher’s exact test for preferential modification (see text) from left-to-right within each row, and top-to-bottom across rows, with the most significant protein at the top left. (PDF 24 kb) [file 12864_2017_3676_MOESM3_ESM.pdf]

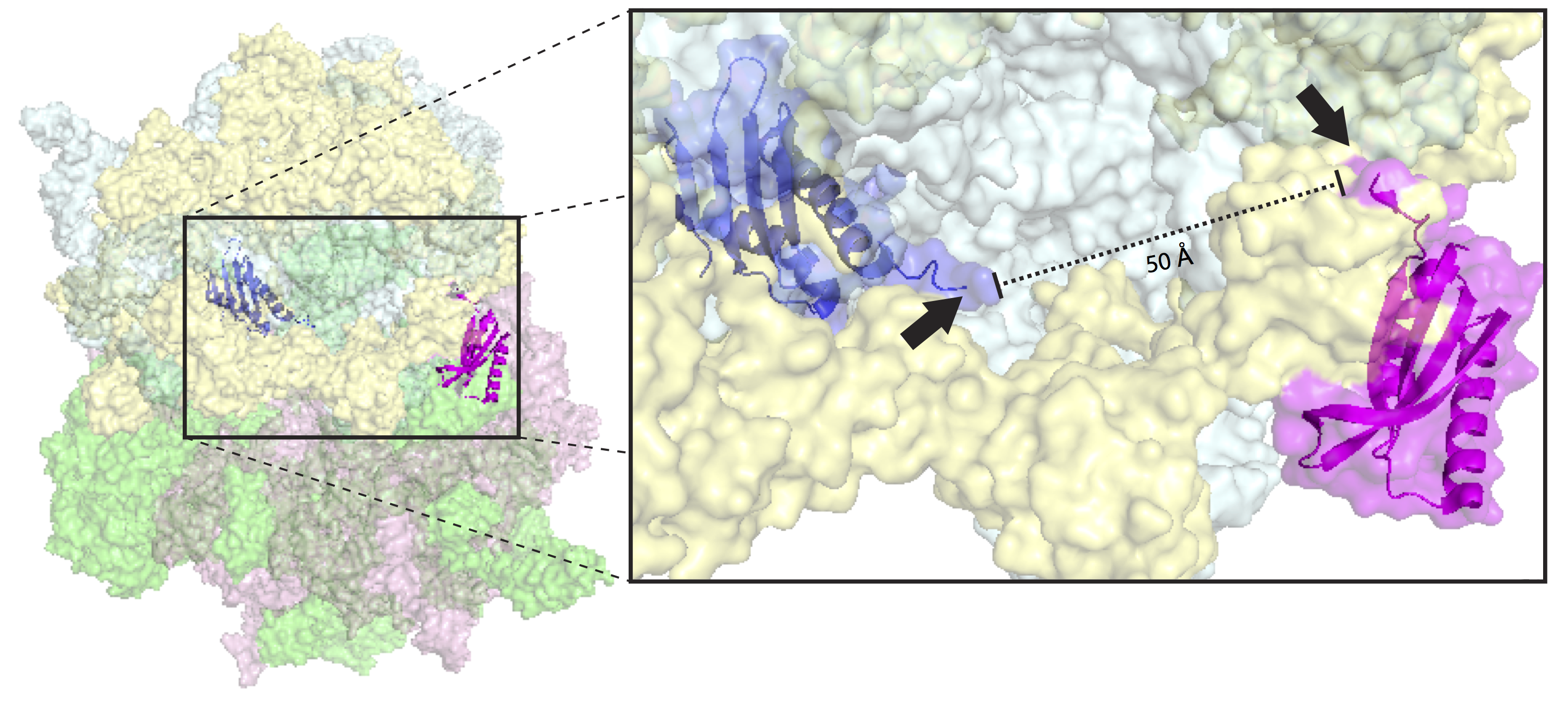

Supplement: Supplementary file 4 — Relative locations of YfiA (blue) and native T. thermophilus S6 (magenta) proteins in crystal structure of E. coli YfiA bound to the T. thermophilus 70S ribosome (PDB ID 4V8I [49]). YfiA is positioned within the 30S subunit mRNA tunnel, and S6 on the outer surface of the 30S subunit; the C-terminal tails of both proteins (black arrows) point toward the same region of the 16S rRNA (light blue). The 17 C-terminal residues for YfiA, including the terminal glutamate residues, were not resolved in the crystal structure; the T. thermophilus S6 protein coding sequence ends at residue 101, lacking the 30-AA unstructured C-terminal domain present in E. coli S6. 16S rRNA is shown in light blue; 30S ribosomal proteins (other than S6) are shown in light yellow; 50S ribosomal proteins are shown in green; and 23S rRNA is shown in pink. (PNG 3630 kb) [file 12864_2017_3676_MOESM4_ESM.png]

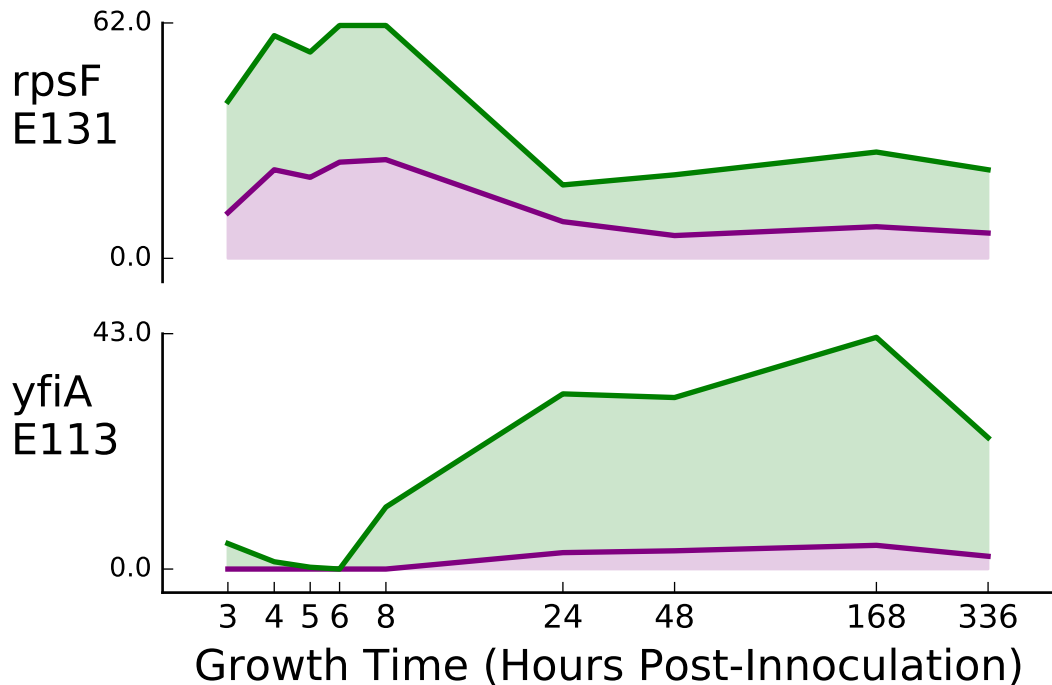

Supplement: Supplementary file 5 — Modified and unmodified PSM counts for each AA position with a C-terminal +129 Da modification across all timepoints. The plots show unmodified (green) and +129 Da modified (purple) PSM counts across all nine timepoints (x-axis) for the C-terminal position of the two proteins that have at least one PSM identified by MODa as containing a C-terminal +129 Da modification. Counts represent the average of the three biologcial replicates. (PDF 14 kb) [file 12864_2017_3676_MOESM5_ESM.pdf]

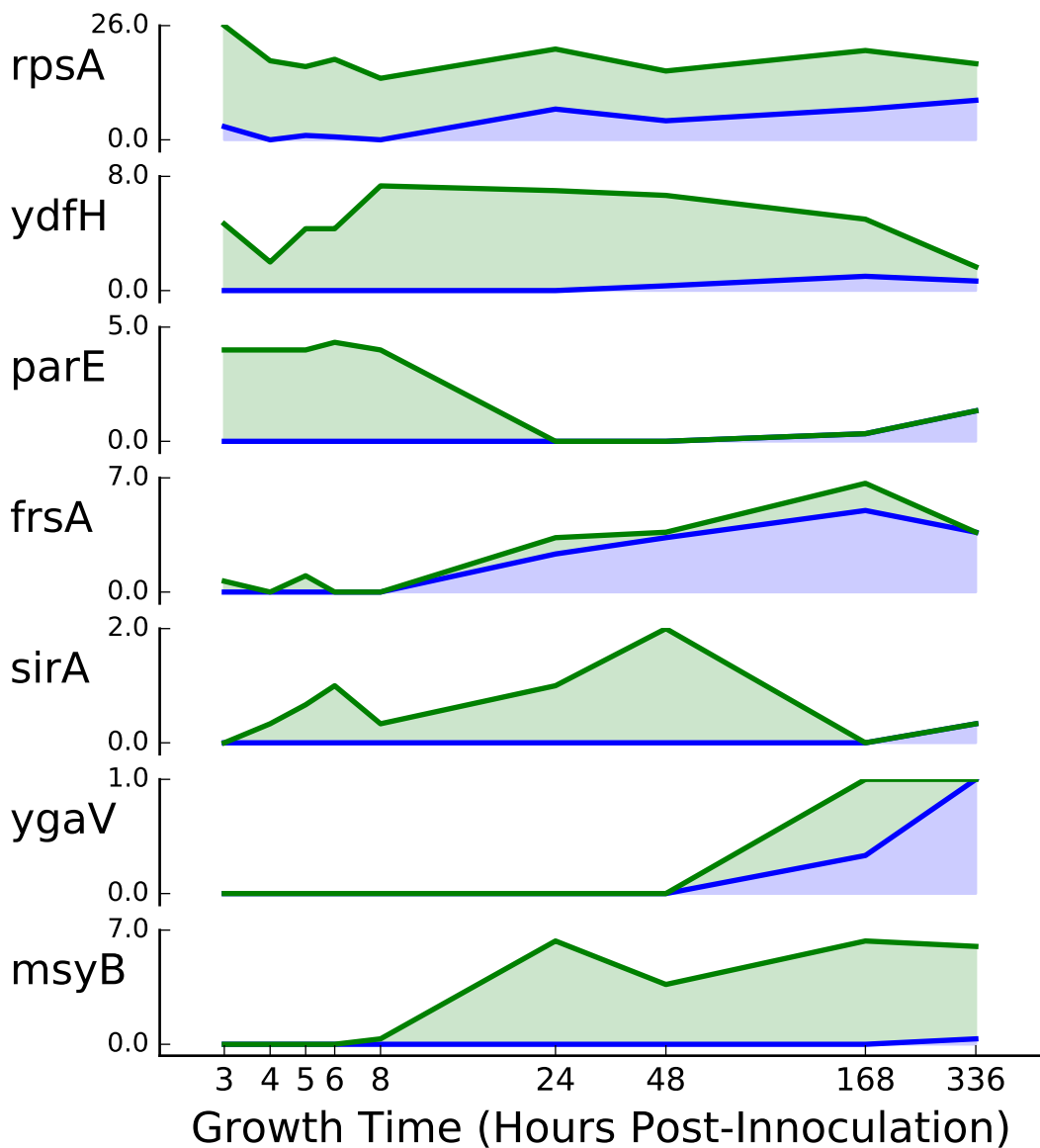

Supplement: Supplementary file 6 — Temporally variable modification for individual proteins with an N-terminal threonine possessing a +42 Da modification. The plots show unmodified (green) and +42 Da Modified (blue) PSM counts across all nine timepoints (x-axis) for the N-terminal position of all proteins that have both (i) at least one PSM identified by MODa as containing an N-terminal +42 Da modification and (ii) having a penultimate threonine (AA position 2; i.e. the N-terminal residue following N-terminal methionine cleavage). Counts represent the average of the three biological replicates. Plots are ordered from top to bottom by the mean p value of the Fisher’s exact test for preferential modification (see text) from top to bottom, with the most significant protein at the top. (PDF 17 kb) [file 12864_2017_3676_MOESM6_ESM.pdf]

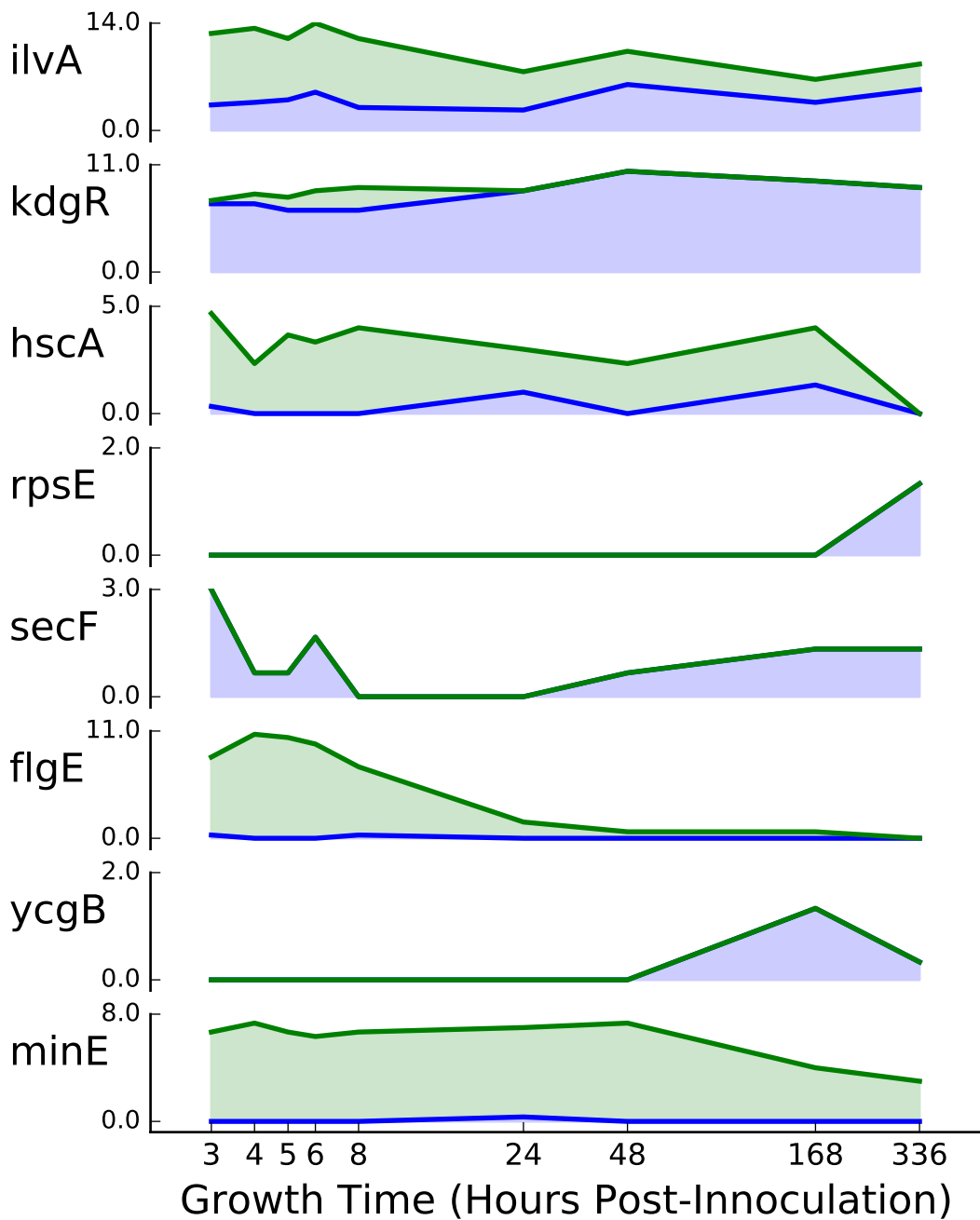

Supplement: Supplementary file 7 — Fraction of total peptides across timepoints with an N-terminal alanine possessing a +42 Da modification. The plots show unmodified (green) and +42 Da modified (blue) PSM counts across all nine timepoints (x-axis) for the N-terminal position of all proteins that have both (i) at least one PSM identified by MODa as containing an N-terminal +42 Da modification and (ii) having a penultimate Alanine (AA position 2; i.e. the N-terminal residue following N-terminal methionine cleavage). Counts represent the average of the three biologcial replicates. Plots are ordered from top to bottom by the mean p value of the Fisher’s exact test for preferential modification (see text) from top to bottom, with the most significant protein at the top. (PDF 17 kb) [file 12864_2017_3676_MOESM7_ESM.pdf]

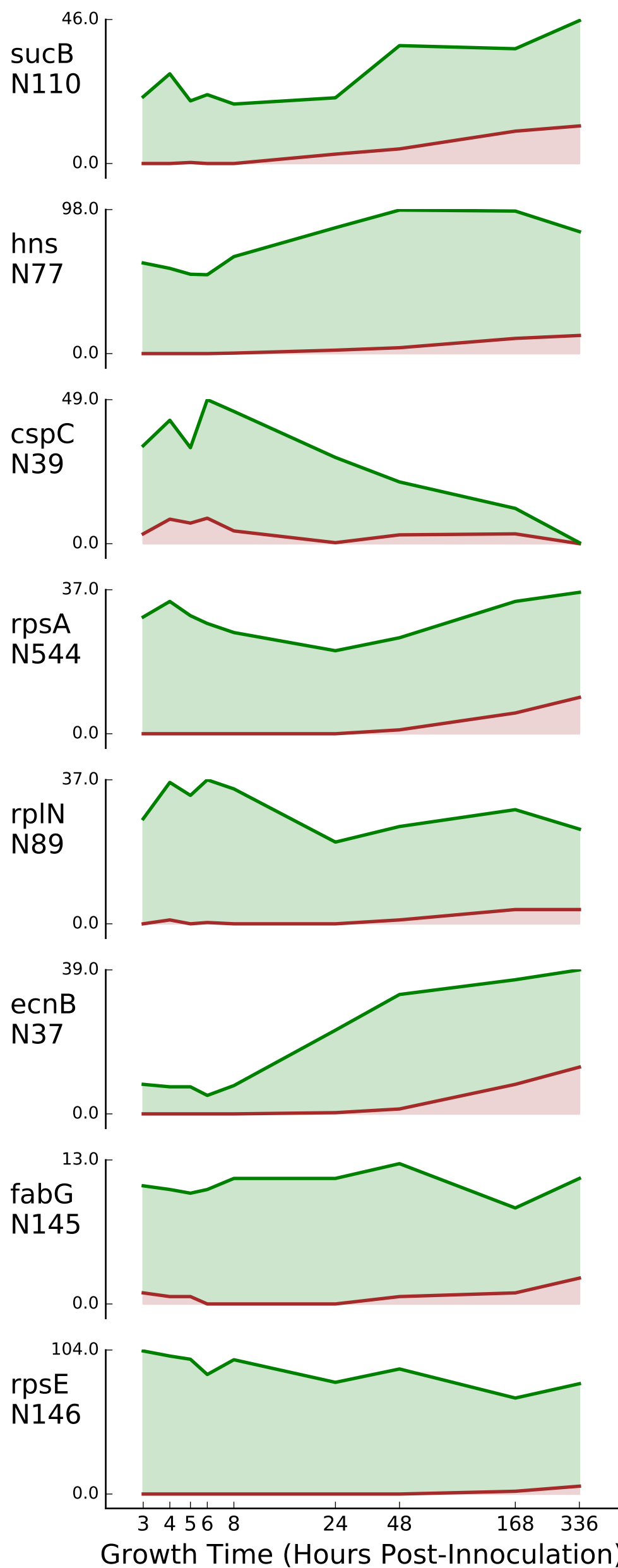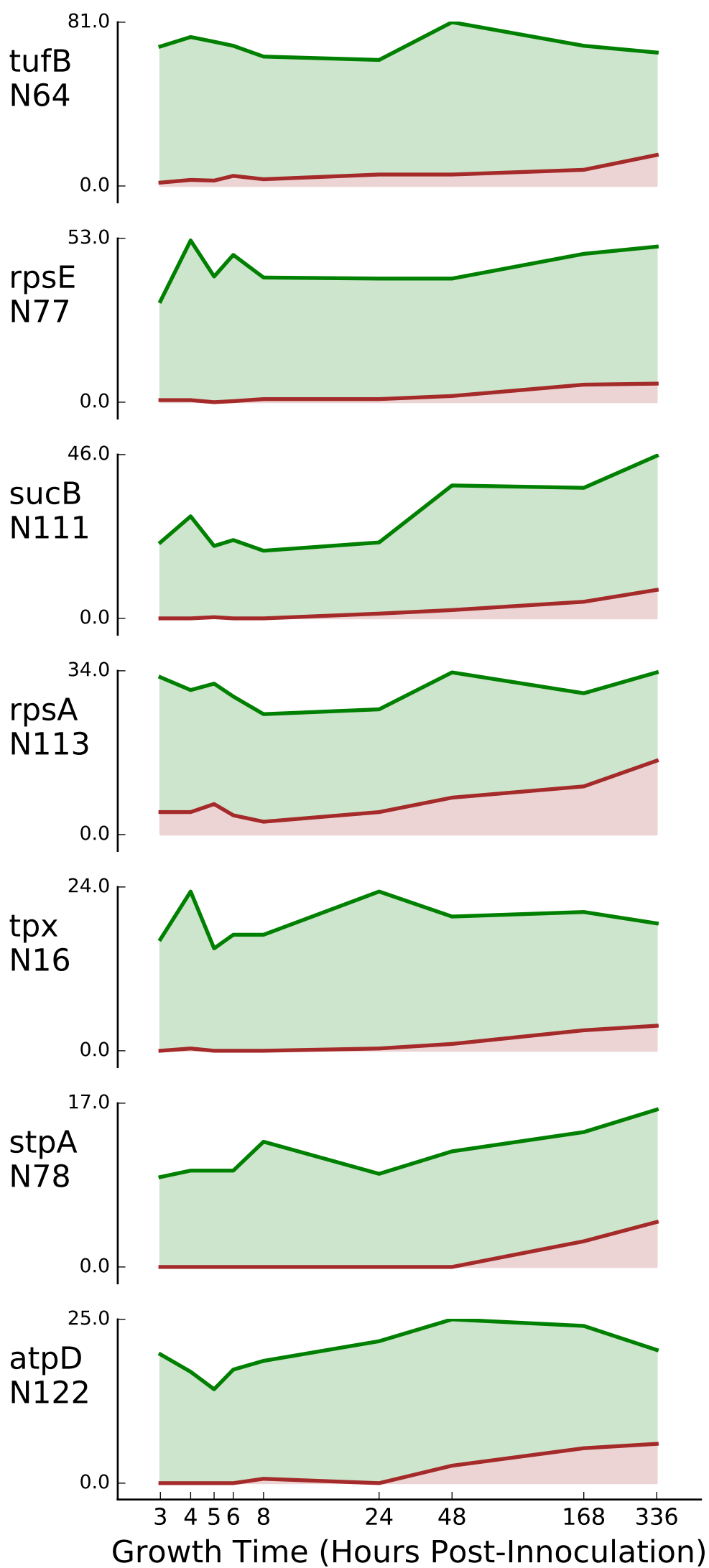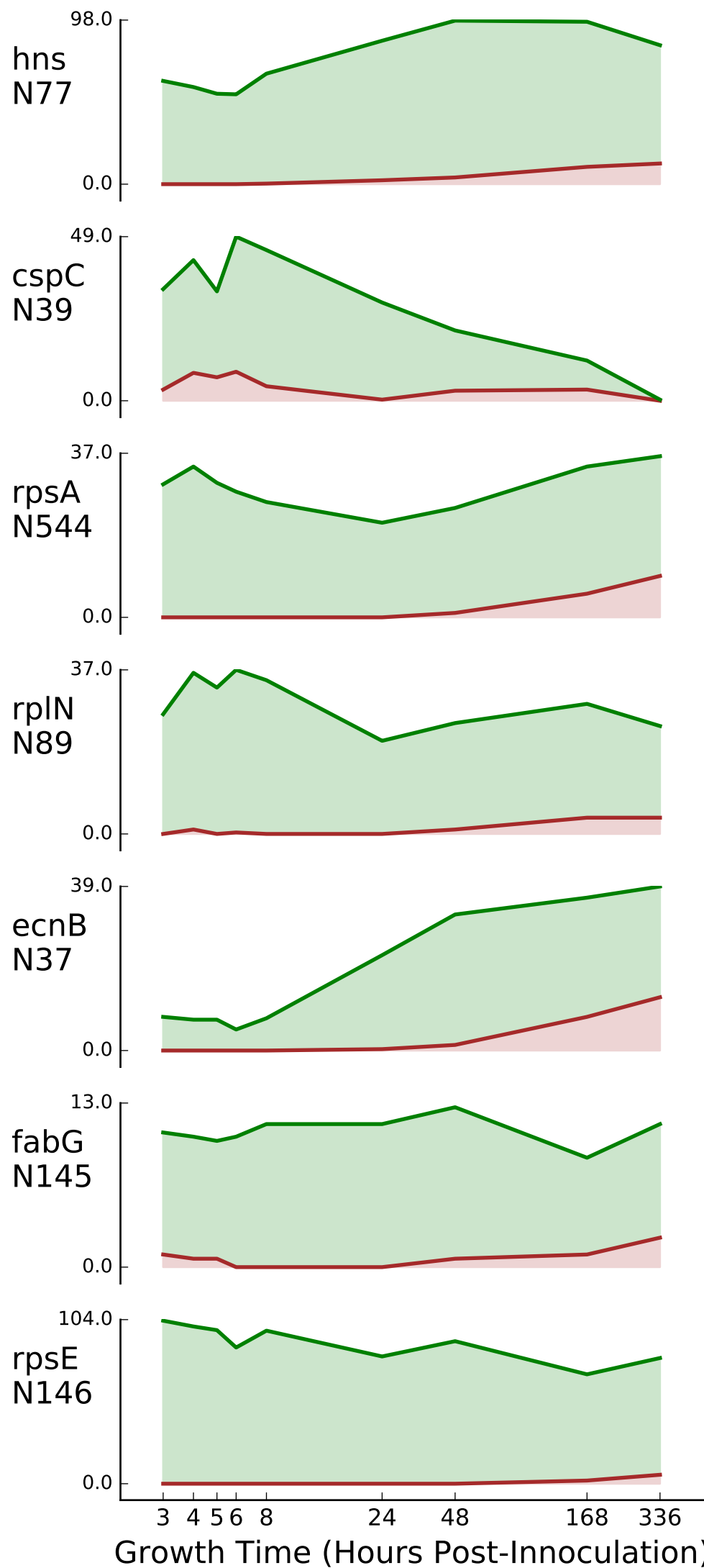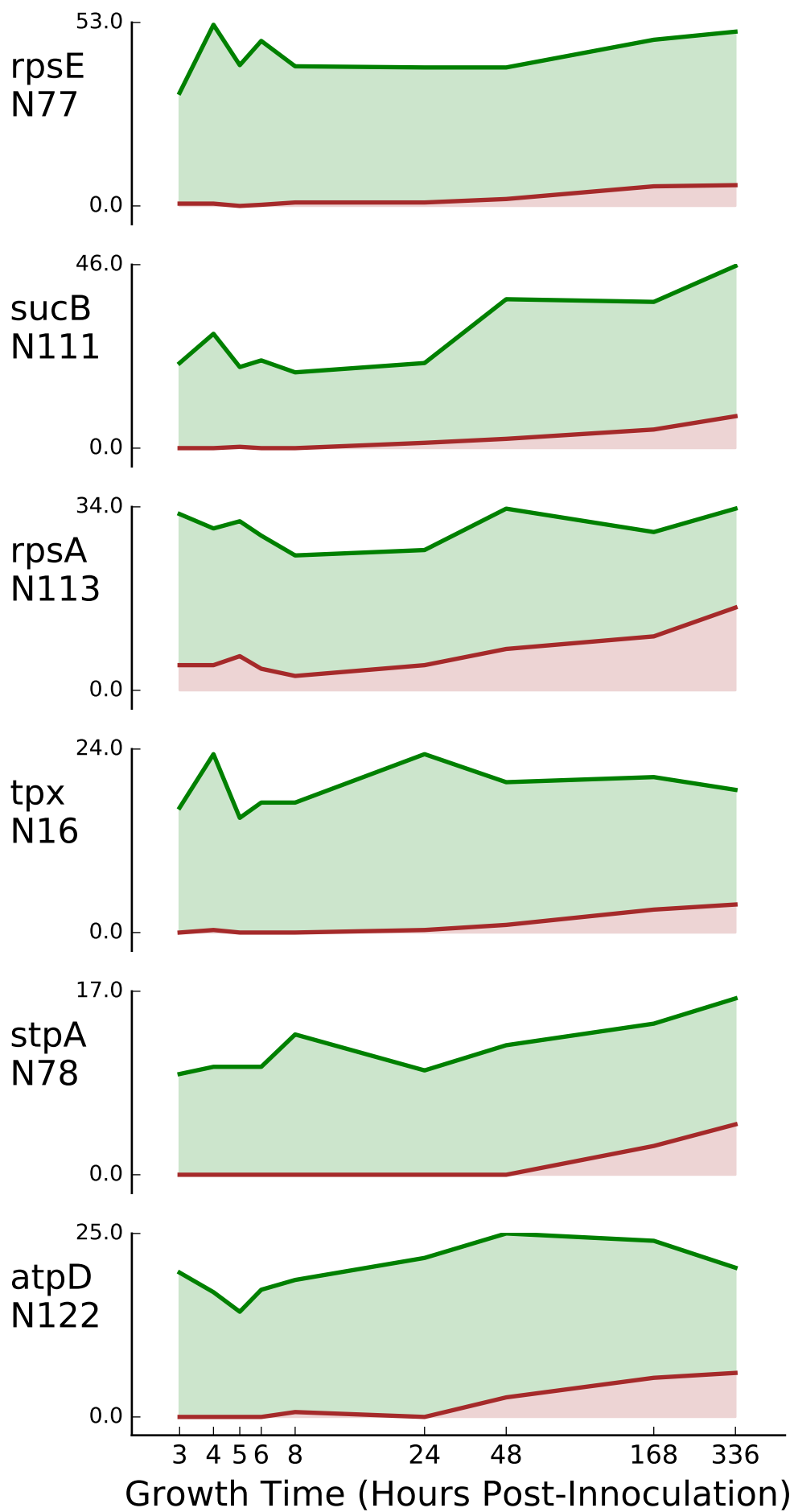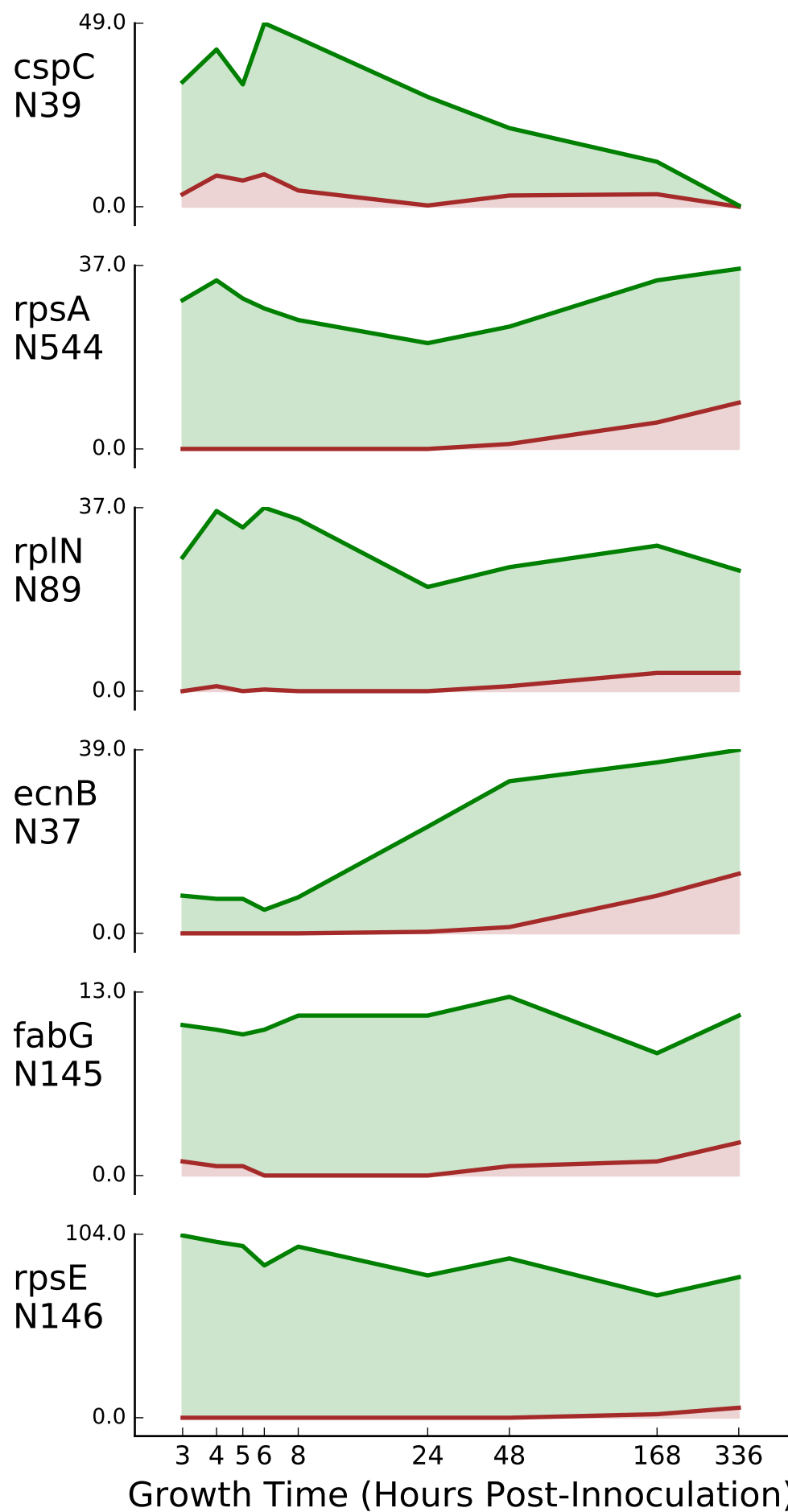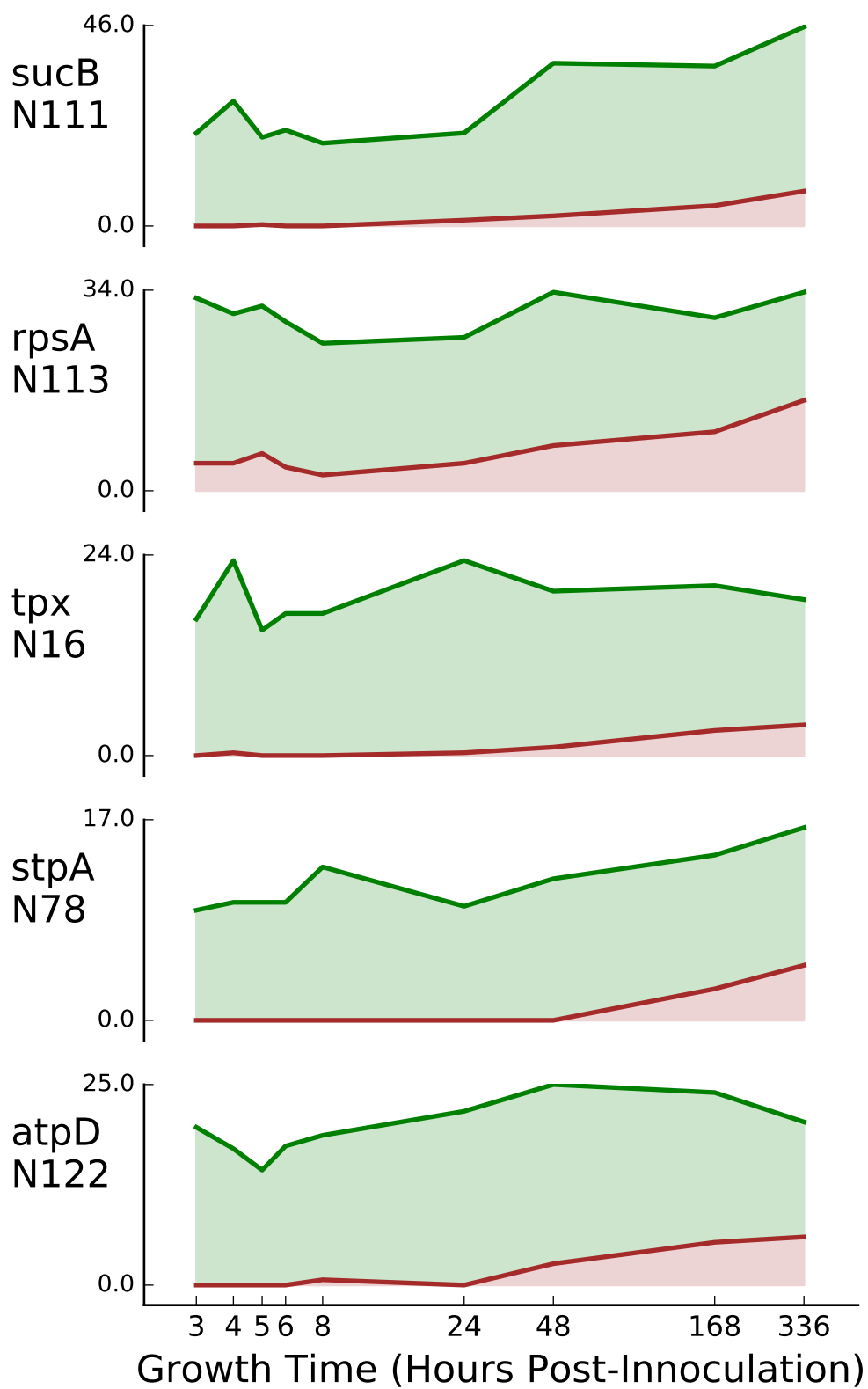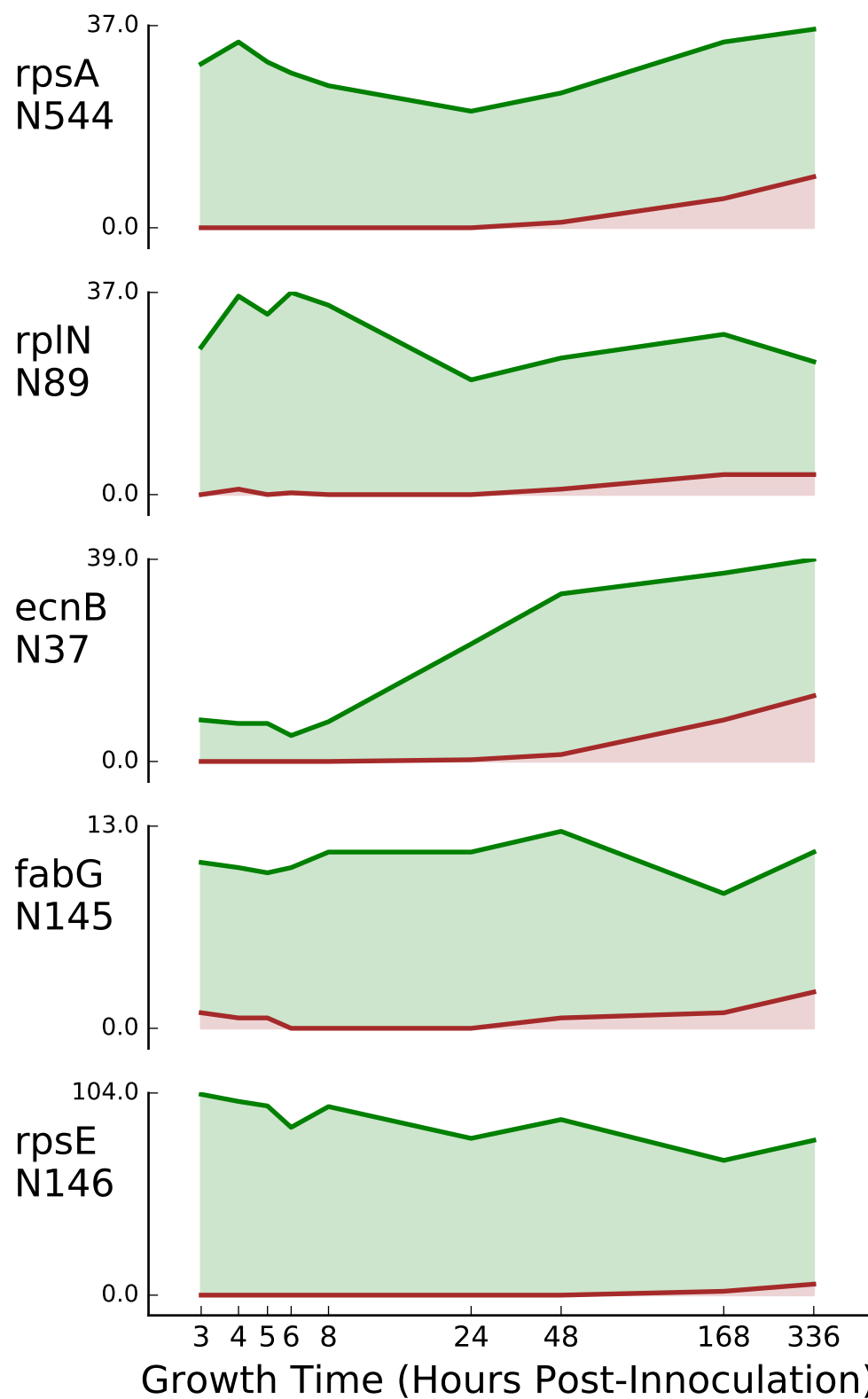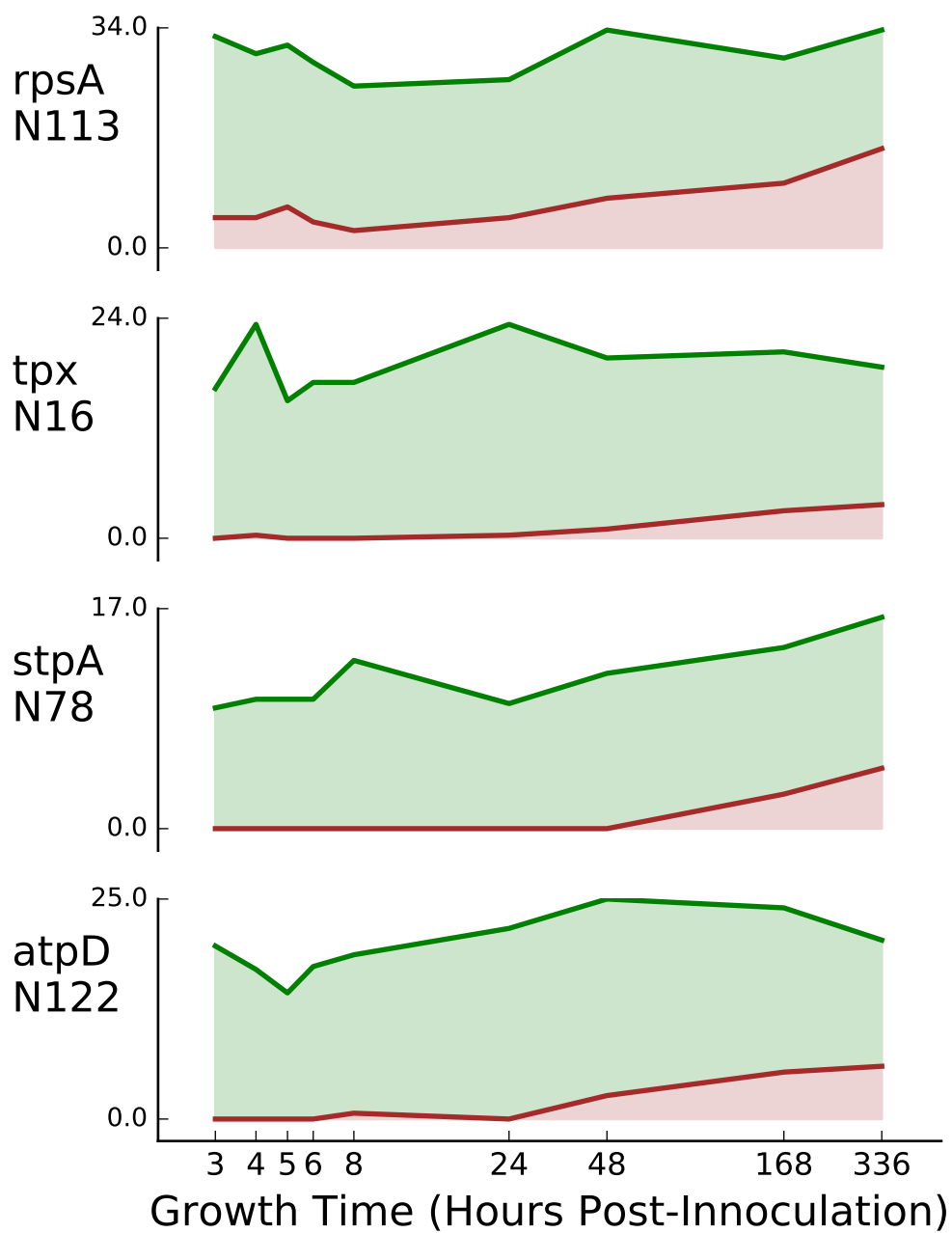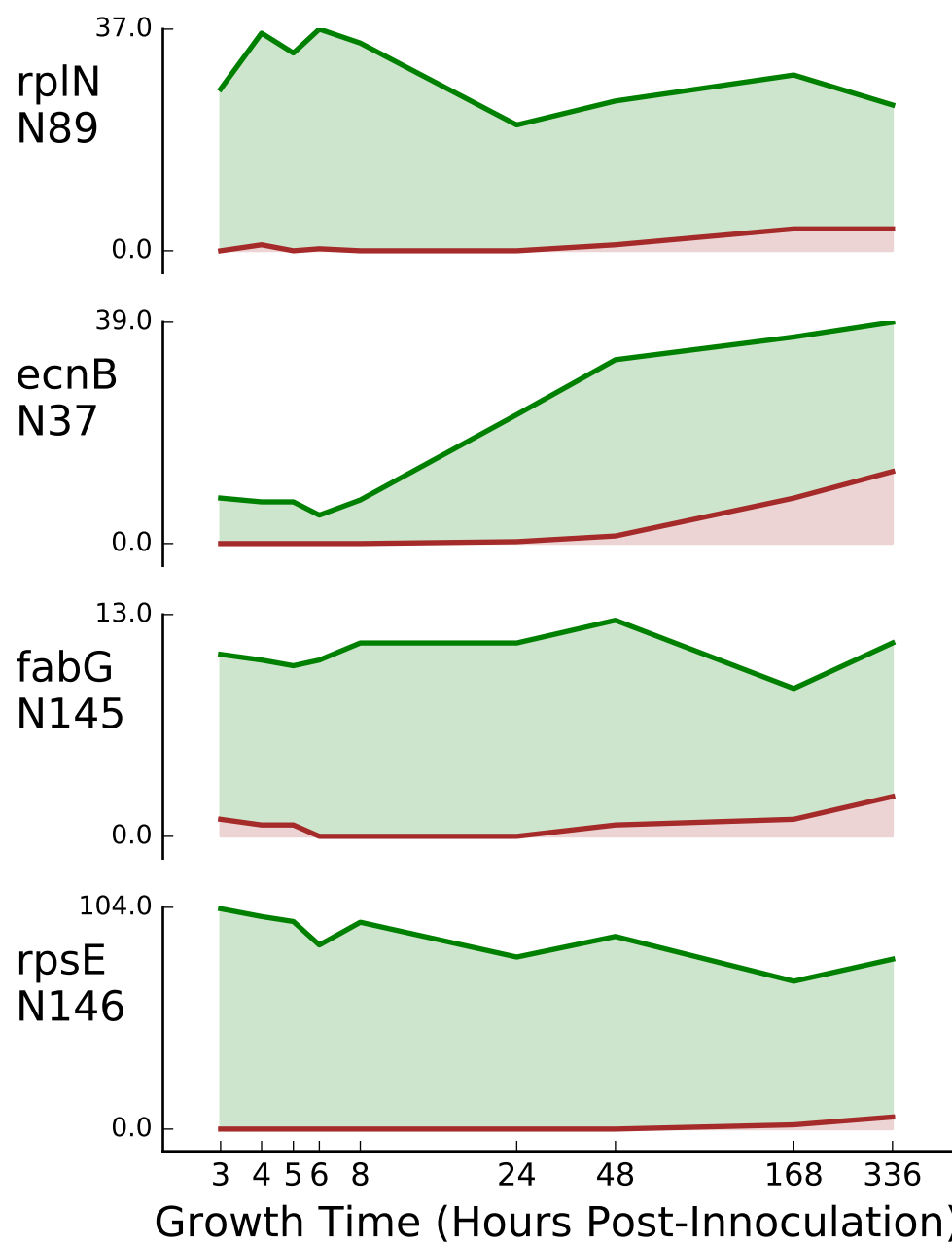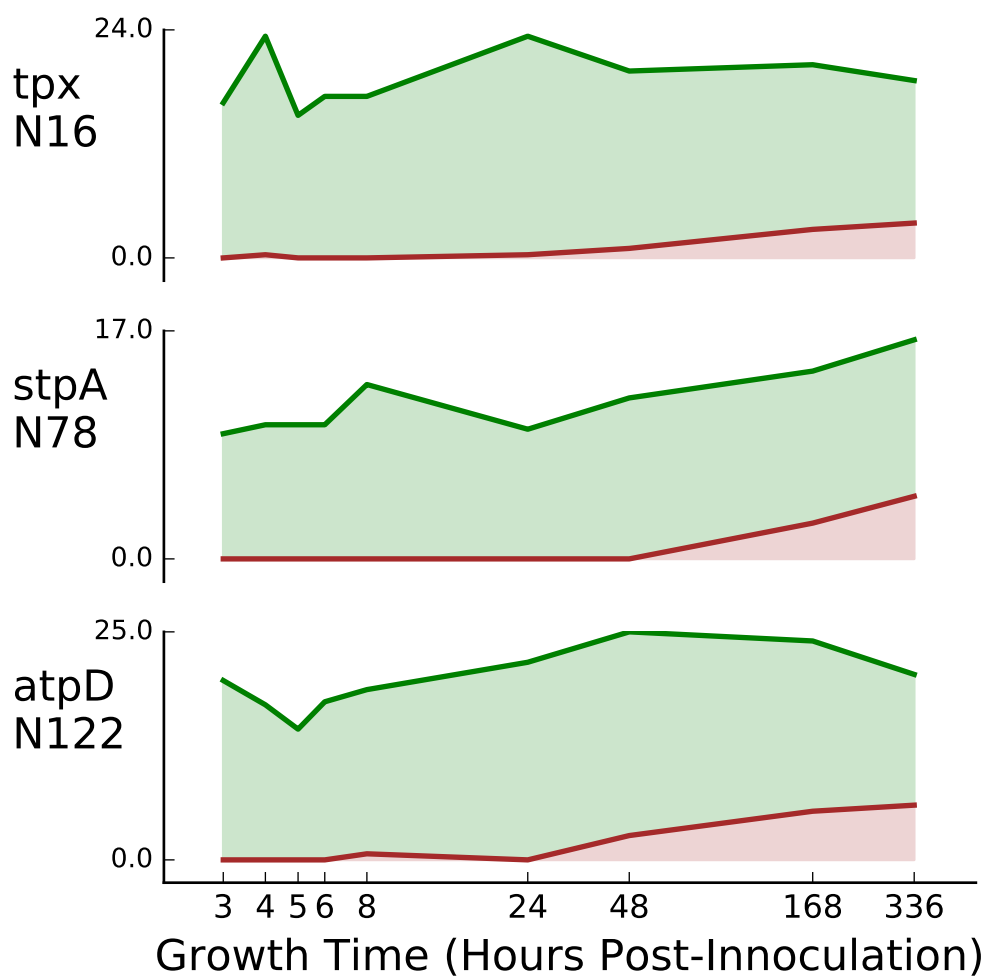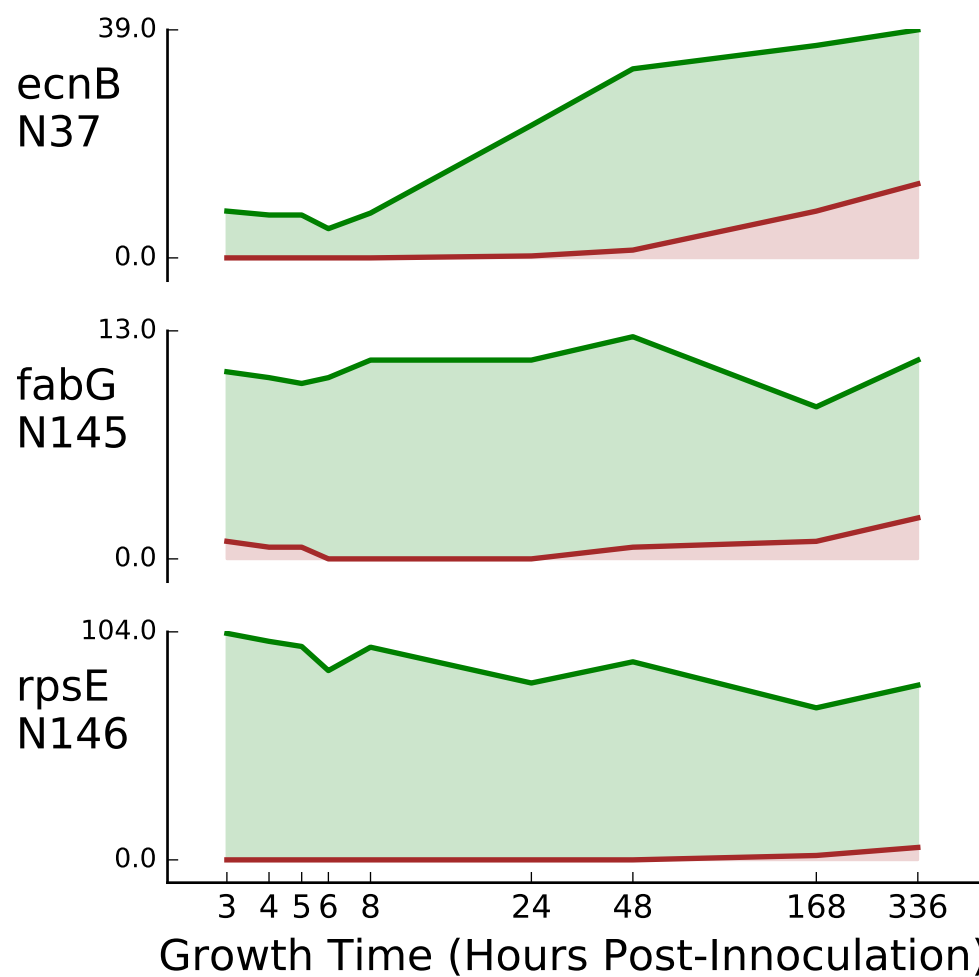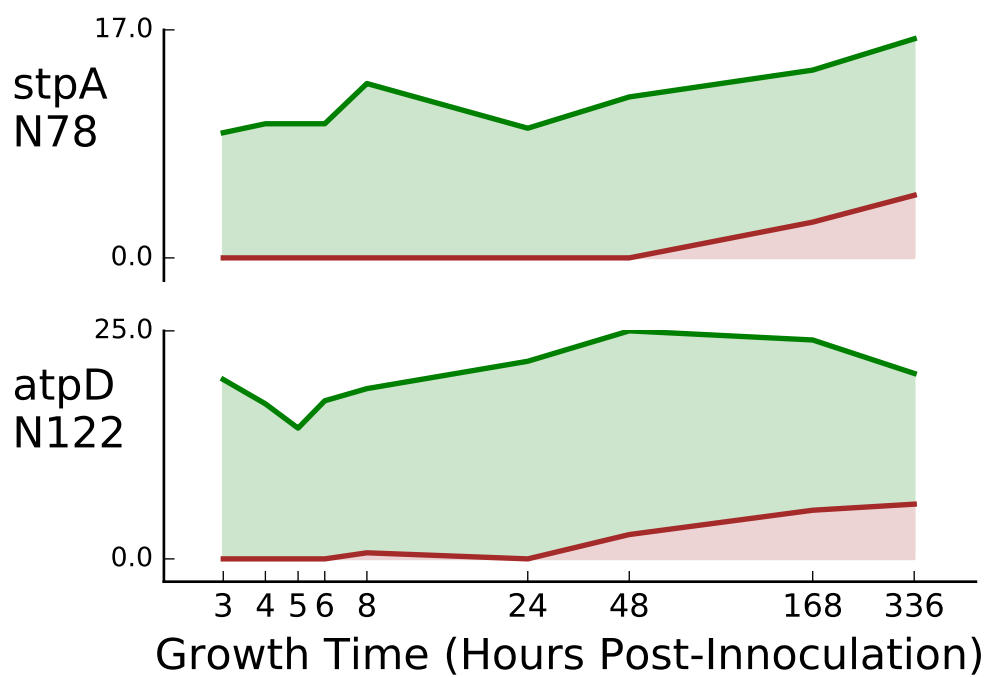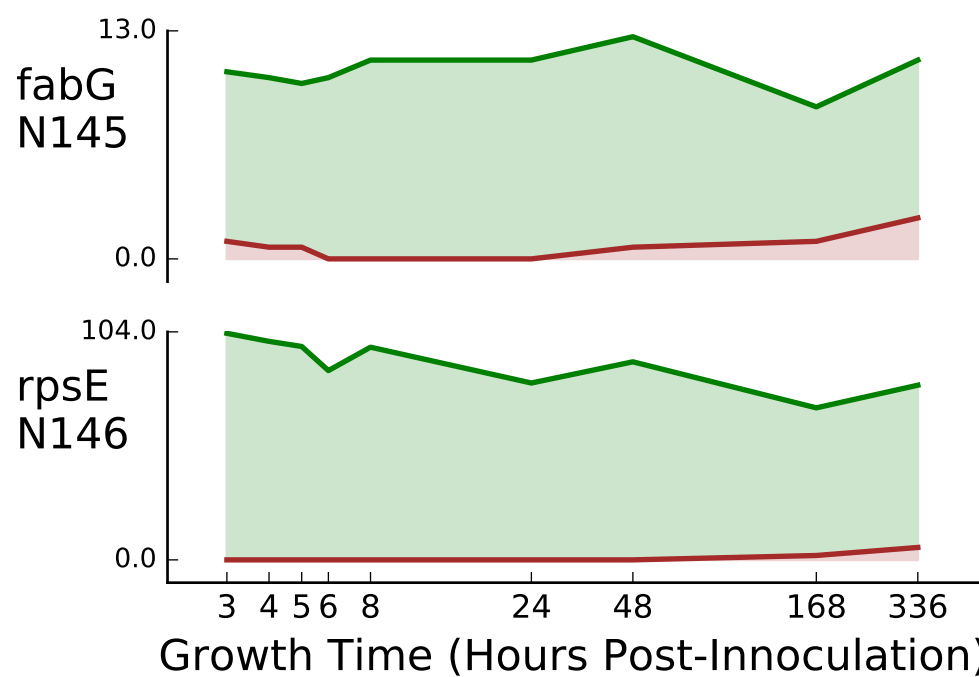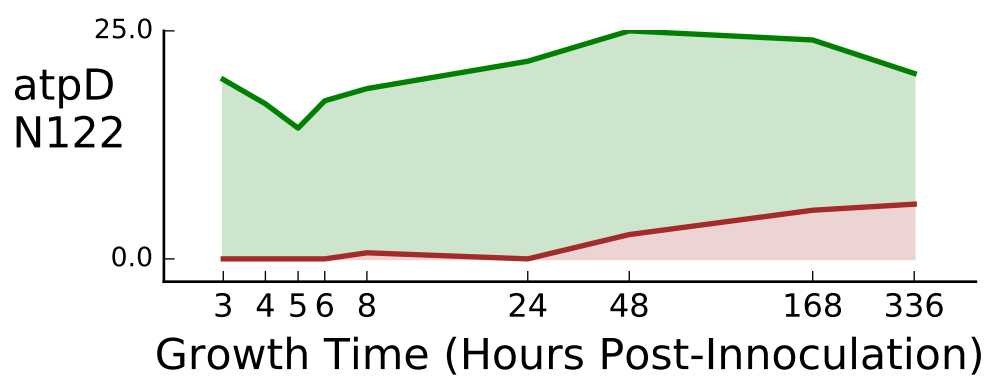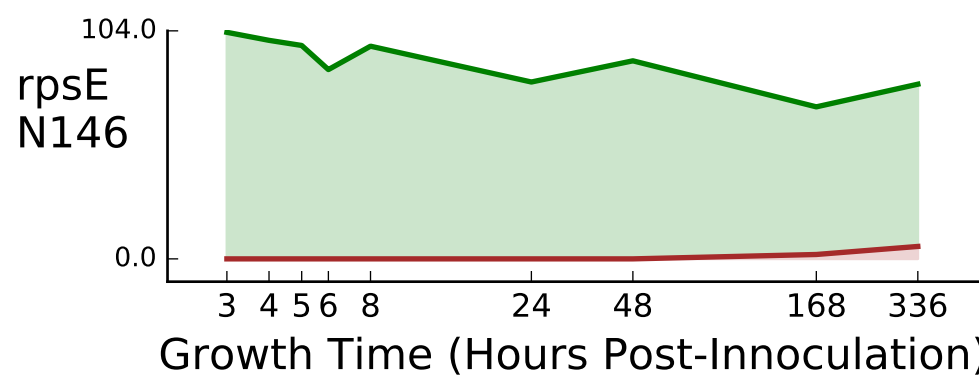

Supplement: Supplementary file 8 — Modified and unmodified PSM counts for each AA position with a significantly stationary-phase biased +1 Da modification to asparagine. The plots show unmodified (green) and +1 Da modified (brown) PSM counts across all nine timepoints (x-axis) for the 10 asparagine residues with the most significant p-values across all three biological replicates. Counts represent the average of the three biologcial replicates. Plots are ordered by the mean p value of the Fisher’s exact test for preferential modification from left-to-right within each row, and from top-to-bottom across rows, with the most significant position at the top left. (PDF 20 kb) [file 12864_2017_3676_MOESM8_ESM.pdf]

A

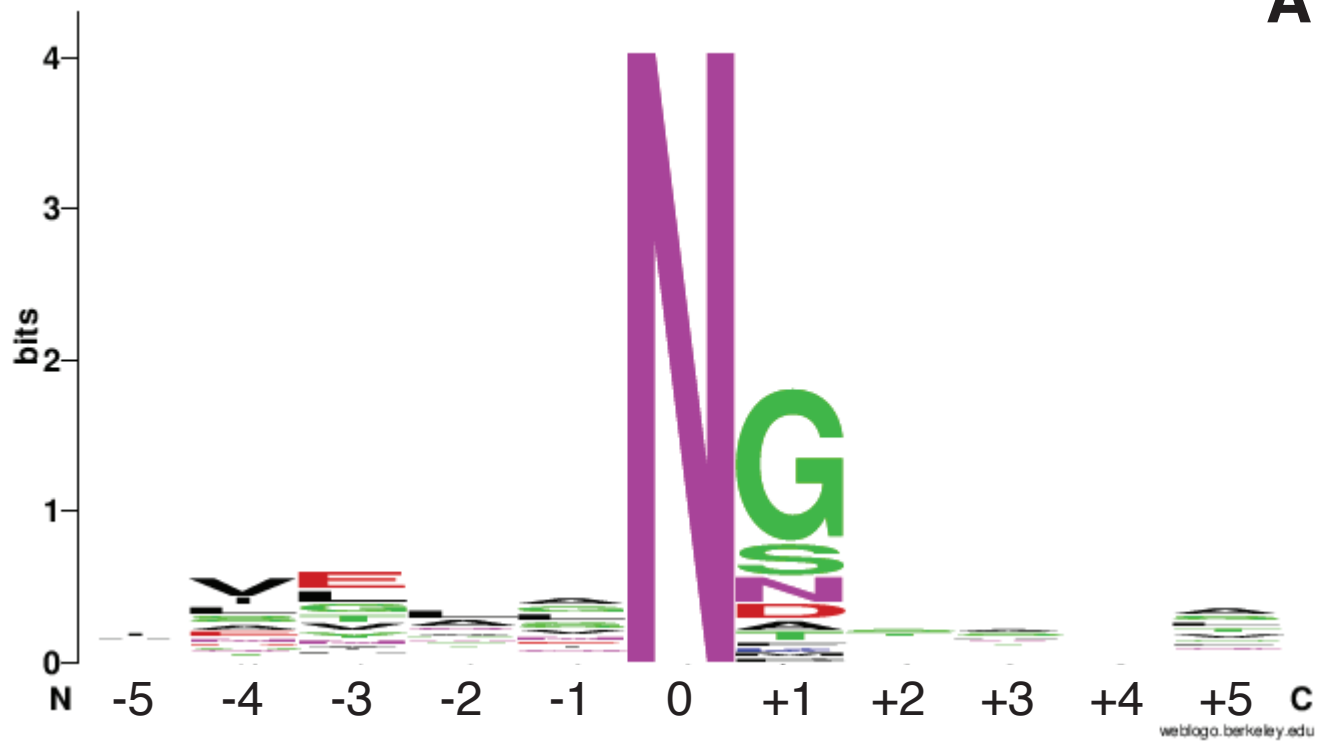

B

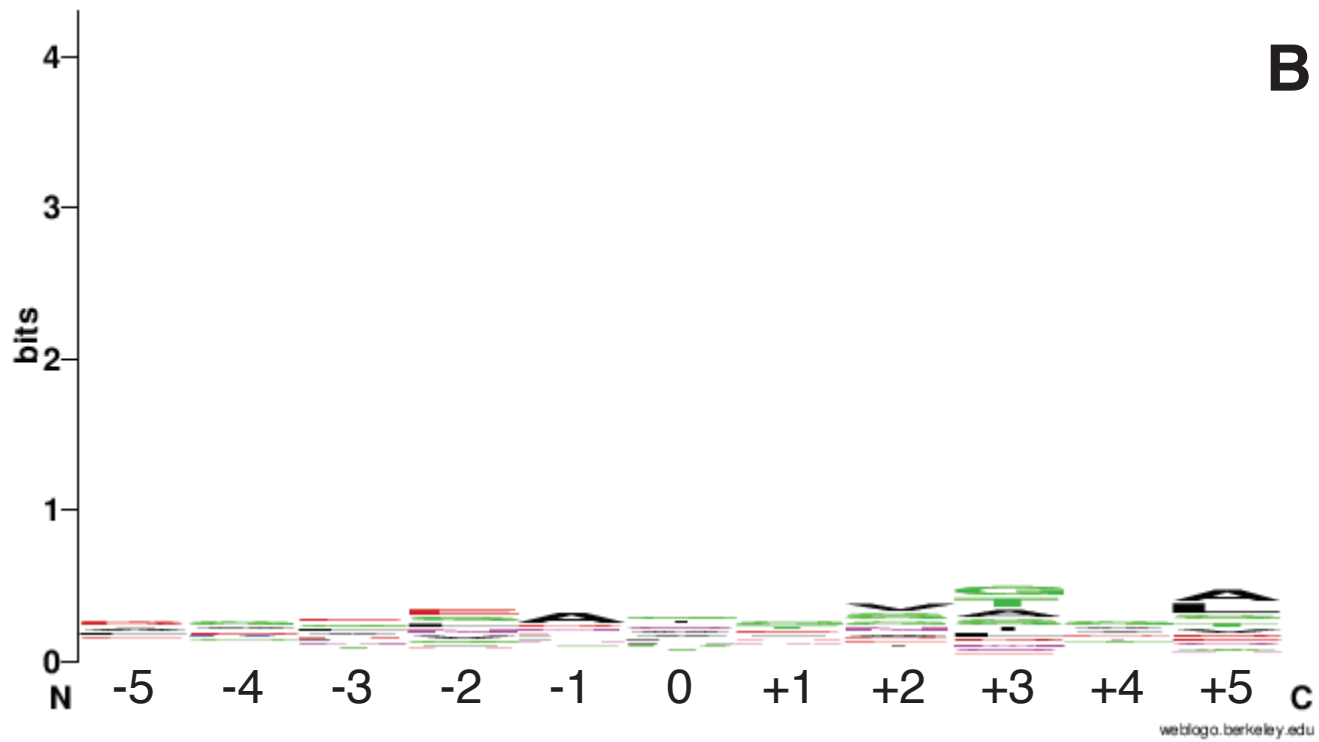

Supplement: Supplementary file 9 — Amino Acid sequence logos generated using WebLogo [92] for a +/- 5 AA window around the MODa-called site of modification for the top 50 most abundant +1Da modifications localized at Asparagine residues (A) and at all other residue types combined (B). Asparagine residues show a preferential enrichment of Glycine, Serine, and Asparagine AAs at the +1 position not observed for non-Asn modifications. (PDF 347 kb) [file 12864_2017_3676_MOESM9_ESM.pdf]

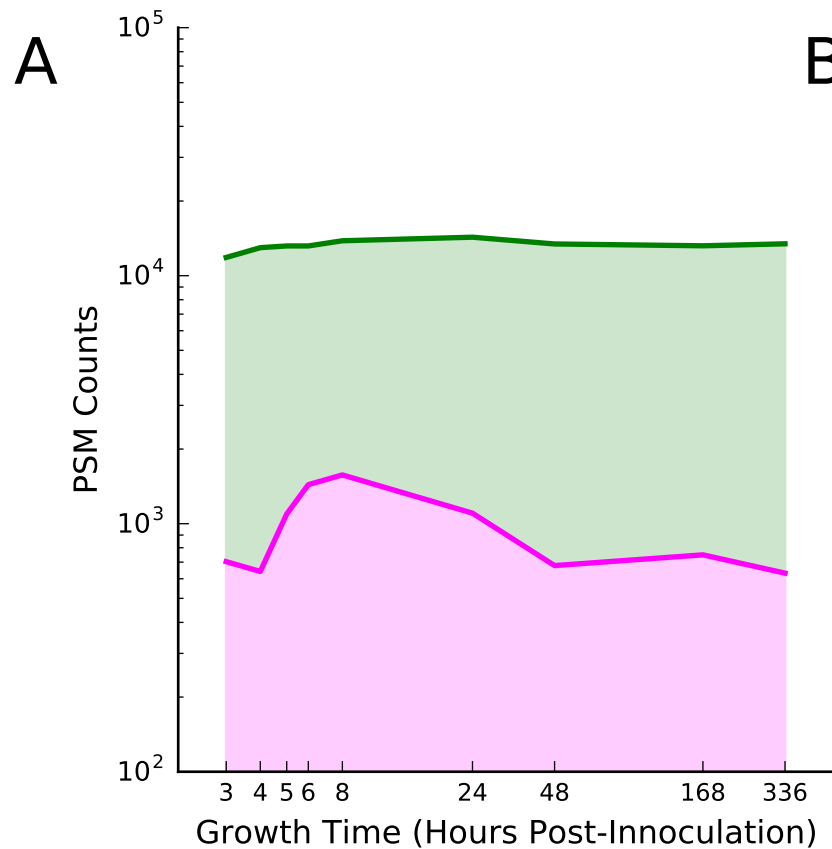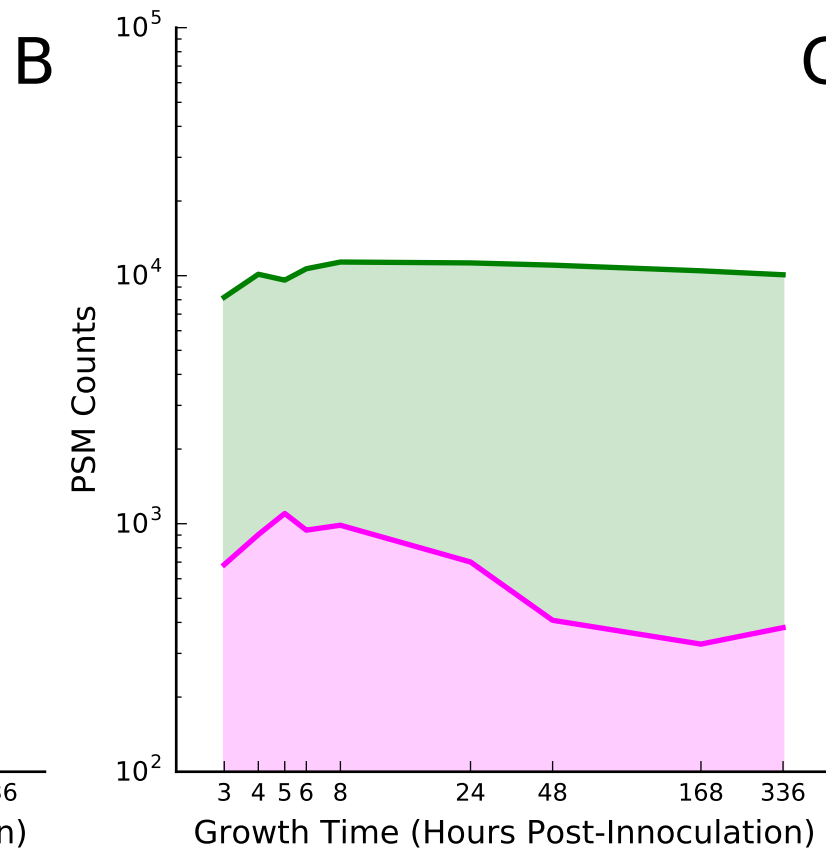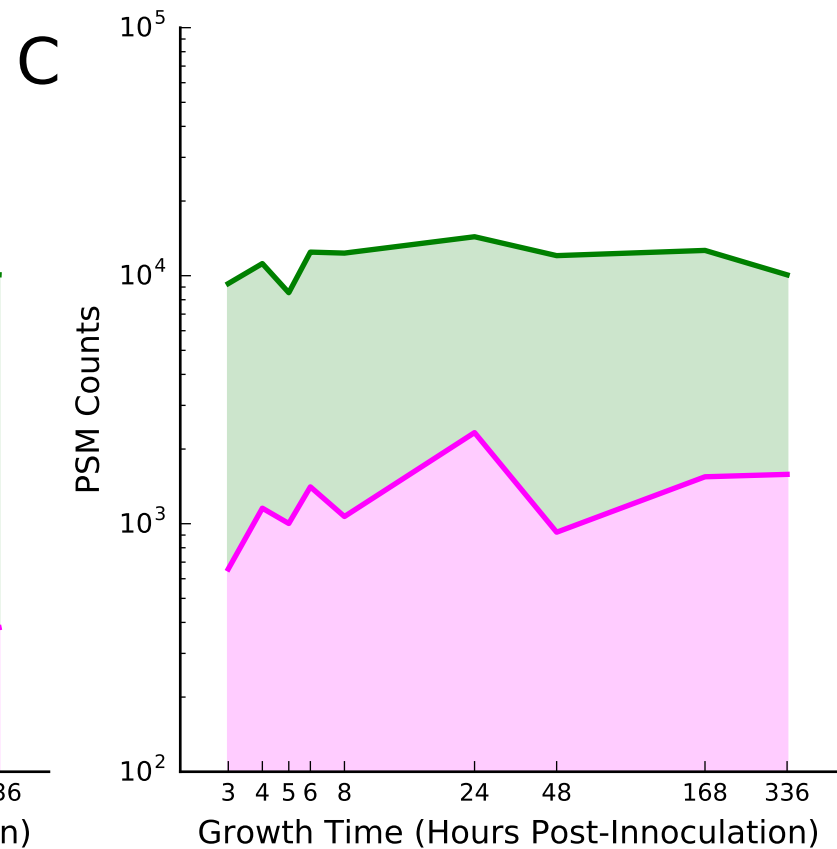

Supplement: Supplementary file 10 — Modified and unmodified counts across timepoints for all AA positions with a +16 Da modification to methionine, pooled by biological replicate. The plots show the total unmodified (green) and +16 Da modified (magenta) PSM counts across all nine timepoints (x-axis) for methionine residues that have at least one +16 Da modification at any time point in any replicate. The three panels show counts for each of the three biological replicates, replicate 1 (A), replicate 2 (B) and replicate 3 (C). Note that the y-axis is plotted on a logarithmic (base 10) scale due to the high number of total counts relative to modified counts. (PDF 16 kb) [file 12864_2017_3676_MOESM10_ESM.pdf]

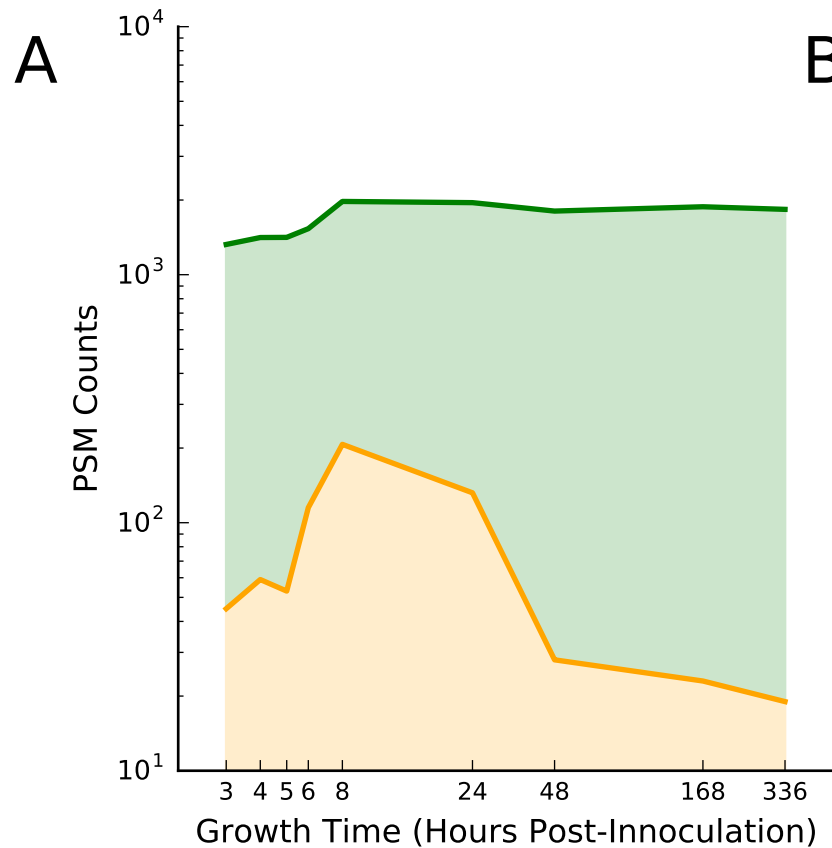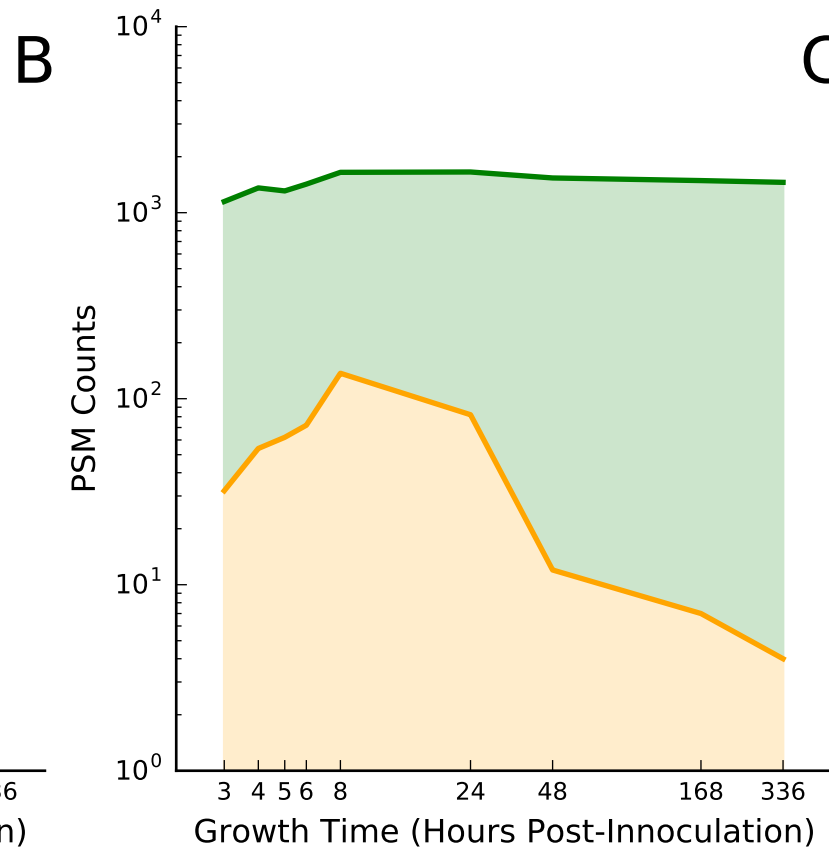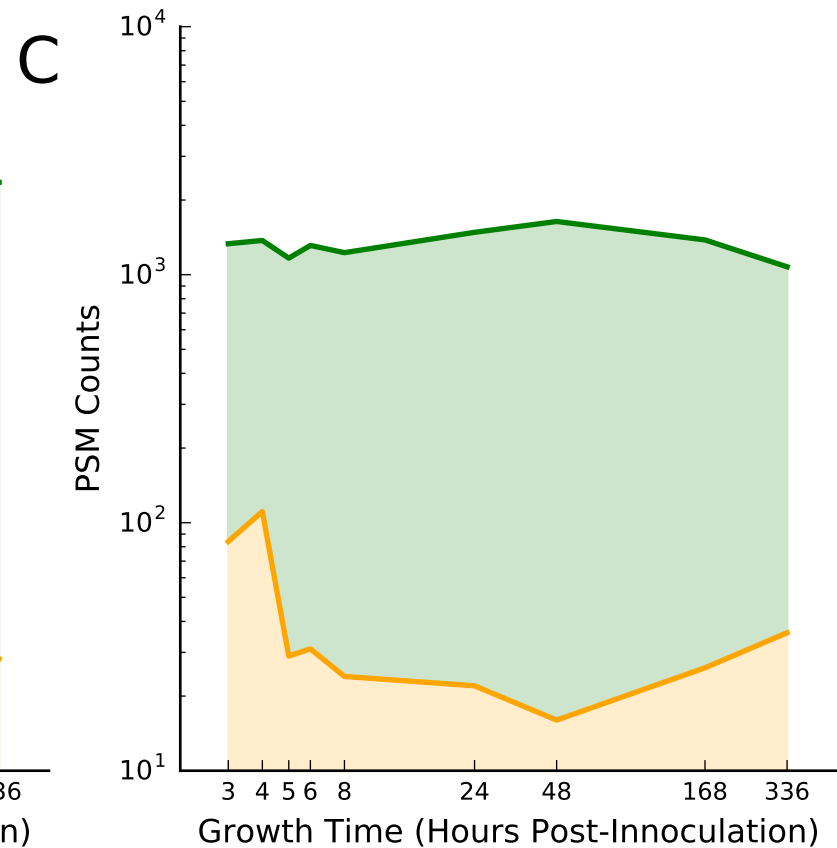

Supplement: Supplementary file 11 — Modified and unmodified counts across timepoints for all AA positions with a +16 Da modification to tryptophan, pooled by biological replicate. The plots show the total unmodified (green) and +16 Da modified (orange) PSM counts across all nine timepoints (x-axis) for tryptophan residues that have at least one +16 Da modification at any time point in any replicate. The three panels show counts for each of the three biological replicates 1 (A), 2 (B) and 3 (C). Note that the y-axis is plotted on a logarithmic (base 10) scale due to the high number of total counts relative to modified counts. (PDF 16 kb) [file 12864_2017_3676_MOESM11_ESM.pdf]

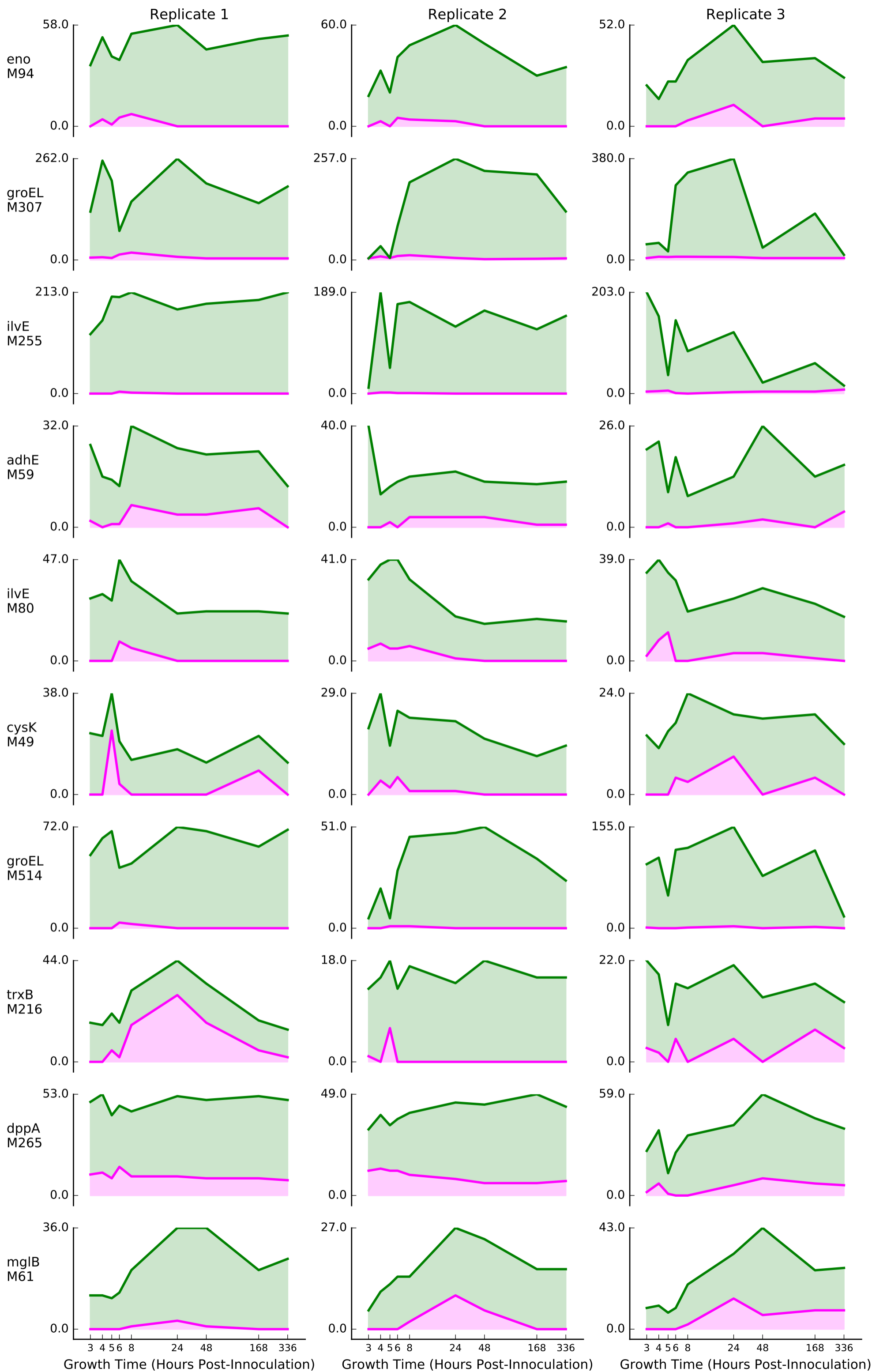

Supplement: Supplementary file 12 — Modified and unmodified counts across timepoints for the top 10 exponential-enriched AA positions with a +16 Da modification to methionine. The plots show unmodified (green) and +16 Da modified (magenta) methionine PSM counts across all nine timepoints (x-axis) for the protein and position indicated. Plots in columns correspond to the three biological replicates 1 (left column), 2 (center column), and 3 (right column). Counts represent the average of the three biologcial replicates. Plots are ordered from top to bottom by the mean p value of the Fisher’s exact test for preferential modification (see text), with the most significant protein at the top. (PDF 25 kb) [file 12864_2017_3676_MOESM12_ESM.pdf]

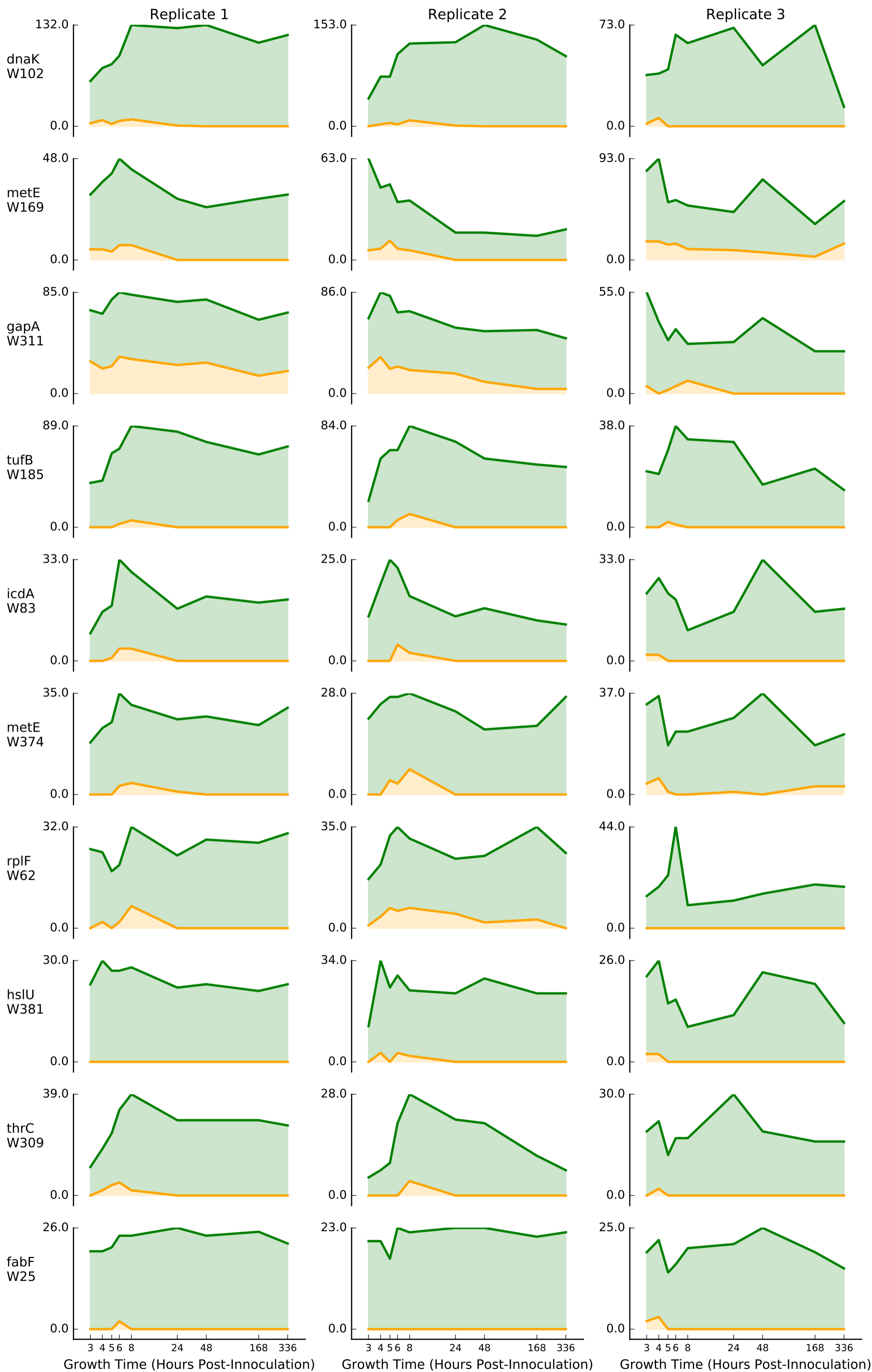

Supplement: Supplementary file 13 — Modified and unmodified counts across timepoints for the top 15 exponential-enriched AA positions with a +16 Da modification to tryptophan. The plots show unmodified (green) and +16 Da modified (orange) tryptophan PSM counts across all nine timepoints (x-axis) for the protein and position indicated. Plots in columns correspond to the three biological replicates 1 (left column), 2 (center column), and 3 (right column). Counts represent the average of the three biologcial replicates. Plots are ordered from top to bottom by the mean p value of the Fisher’s exact test for preferential modification (see text), with the most significant protein at the top. (PDF 25 kb) [file 12864_2017_3676_MOESM13_ESM.pdf]
